# Supplementary material for: Discovering a mitochondrion-localized BAHD acyltransferase involved in calystegine biosynthesis and engineering the production of 3β-tigloyloxytropane
Source: Nat Commun. 2024 Apr 29;15:3623. doi: 10.1038/s41467-024-47968-0 (PMC11058270; doi:10.1038/s41467-024-47968-0)
Supplement: Supplementary file 1 — Supplementary information [file 41467_2024_47968_MOESM1_ESM.pdf]

Discovering a mitochondrion-localised BAHD acyltransferase involved in calystegine biosynthesis and engineering the production of 3*β*-tigloyloxytropane

Junlan Zeng[1], Xiaoqiang Liu[1], Zhaoyue Dong[2], Fangyuan Zhang[1], Fei Qiu[1], Mingyu Zhong[1], Tengfei Zhao[1], Chunxian Yang[1], Lingjiang Zeng[1], Xiaozhong Lan[3], Hongbo Zhang[4], Junhui Zhou[5], Min Chen[2], Kexuan Tang[1,6], Zhihua Liao[1*]

[1] Integrative Science Center of Germplasm Creation in Western China (CHONGQING) Science City, State Key Laboratory of Silkworm Genome Biology, SWU-TAAHC Medicinal Plant Joint R&D Centre, School of Life Sciences, Southwest University, Chongqing 400715, China

[2] College of Pharmaceutical Sciences, Southwest University, Chongqing 400715, China

[3] TAAHC-SWU Medicinal Plant Joint R&D Centre, The Provincial and Ministerial Co-founded Collaborative Innovation Center for R&D in Xizang Characteristic Agricultural and Animal Husbandry Resources, Xizang Agricultural and Animal Husbandry College, Nyingchi of Xizang 860000, China

[4] Key Laboratory of Synthetic Biology of Ministry of Agriculture and Rural Affairs, Tobacco Research Institute of Chinese Academy of Agricultural Sciences, Qingdao 266101, China

[5] State Key Laboratory of Dao-di Herbs, National Resource Center for Chinese Materia Medica, China Academy of Chinese Medical Sciences, Beijing 100700, China

[6] Fudan-SJTU-Nottingham Plant Biotechnology R&D Center, School of Agriculture and Biology, Shanghai Jiao Tong University, Shanghai 200240, China

*Correspondence: Zhihua Liao (zhliao@swu.edu.cn)

This supplementary information file includes:

Supplementary Table S1. Primers used in this study.

Supplementary Table S2. Gradient profile of mobile phase for LC-MS analyses of 3*β*-acetoxytropane, hygrine, tropinone, 3*α*-tropanol and 3*β*-tropanol.

Supplementary Table S3. Gradient profile of mobile phase for LC-MS analyses of littorine, hyoscyamine, scopolamine, 3*β*-tigloyloxytropane, 3*β*-benzoyloxytropane, tigloyl norpseudotropine, tigloyl 1-hydroxynorpseudotropine and calystegine A3.

Supplementary Fig. S1. The MS/MS spectrum of Tigloyl-CoA.

Supplementary Fig. S2. The MS/MS spectrum of 3β-tigloyloxytropane.

Supplementary Fig. S3. The MS/MS spectrum of 3β-acetoxytropane.

Supplementary Fig. S4. The MS/MS spectrum of 3β-benzoyloxytropane.

Supplementary Fig. S5. The ^{1}H–nuclear magnetic resonance (NMR) spectrum of tigloyl-CoA.

Supplementary Fig. S6. The ^{13}C-NMR spectrum of tigloyl-CoA.

Supplementary Fig. S7. The distortionless enhancement by polarization transfer (DEPT) 135 NMR spectrum of tigloyl-CoA.

Supplementary Fig. S8. The heteronuclear single quantum coherence (HSQC) NMR spectrum of tigloyl-CoA.

Supplementary Fig. S9. The heteronuclear multiple quantum coherence (HMBC) NMR spectrum of tigloyl-CoA.

Supplementary Fig. S10. The ^{1}H–nuclear magnetic resonance (NMR) spectrum of 3β-tigloyloxytropane.

Supplementary Fig. S11. The ^{13}C-NMR spectrum of 3β-tigloyloxytropane.

Supplementary Fig. S12. The distortionless enhancement by polarization transfer (DEPT) 135 NMR spectrum of 3β-tigloyloxytropane.

Supplementary Fig. S13. The heteronuclear single quantum coherence (HSQC) NMR spectrum of 3β-Tigloyloxytropane.

Supplementary Fig. S14. The heteronuclear multiple quantum coherence (HMBC) NMR spectrum of 3β-Tigloyloxytropane.

Supplementary Fig. S15. The ^{1}H-NMR spectrum of 3β-acetoxytropane.

Supplementary Fig. S16. The ^{13}C-NMR spectrum of 3β-acetoxytropane.

Supplementary Fig. S17. The DEPT 135 NMR spectrum of 3β-acetoxytropane.

Supplementary Fig. S18. The HSQC NMR spectrum of 3β-acetoxytropane.

Supplementary Fig. S19. The HMBC NMR spectrum of 3β-acetoxytropane.

Supplementary Fig. S20. The ^{1}H-NMR spectrum of 3β-benzoyloxytropane.

Supplementary Fig. S21. The ^{13}C-NMR spectrum of 3β-benzoyloxytropane.

Supplementary Fig. S22. The DEPT 135 NMR spectrum of 3β-benzoyloxytropane.

Supplementary Fig. S23. The HSQC NMR spectrum of 3β-benzoyloxytropane.

Supplementary Fig. S24. The HMBC NMR spectrum of 3β-benzoyloxytropane.

Supplementary Fig. S25. The analysis of optimum pH and temperature of TS. (A) The optimum pH analysis. (B) The optimum temperature analysis. Recombinant protein obtained from three independent transformants of TS for activity test. The data are presented as means values +/− s.d.

Supplementary Fig. S26. TS catalyses the condensation between 3β-tropanol and acetyl-CoA to generate 3β-acetoxytropane. (A) TS enzymatic assays with acetyl-CoA as acyl donor. (B) Mass spectrometry data of 3β-acetoxytropane.

Supplementary Fig. S27. TS catalyses the condensation between 3β-tropanol and benzoyl-CoA to generate 3β-benzoyloxytropane. (A) TS enzymatic assays with benzoyl-CoA as acyl donor. (B) Mass spectrometry data of 3β-benzoyloxytropane.

Supplementary Fig. S28. The influence of silencing TS on the content of compounds involved in the competitive metabolic flow of TS in *A. belladonna* seedlings. (A) 3α-Tropanol. $^{**}P < 0.0001$. (B) Tropanol hexoside. $^{**}P < 0.0001$. (C) Littorine. (D) Hyoscyamine. (E) Scopolamine. Fifteen independent plants were used in the VIGS assays. Center line of box plot denotes the median value; lower and upper bounds of box plot denote first and third quartile; whiskers of box plot extend to the smallest and maximum values. Statistical analysis was performed according to the two-sided independent sample *t*-test. DW, dry weight.

Supplementary Fig. S29. Plant genomic PCR detection for transgenic hairy root cultures. M, DNA marker. P, plasmid pBI121-TS, used as positive control. N, plasmid pBI121, used as negative control. CK, root cultures lines transformed by pBI121. TS, all independently transformed root cultures lines

with overexpression of TS (three biological replicates for each line), including OE-1, OE-2, OE-3, and OE-4.

Supplementary Fig. S30. The influence of TS overexpression on the content of compounds in the competitive metabolic flow of TS in *A. belladonna* hairy root cultures. (A) 3*α*-Tropanol. (B) Tropanol hexoside. (C) Littorine. (D) Hyoscyamine. (E) Scopolamine. OE denotes all independently transformed root culture lines overexpressing TS (three biological replicates for each line), including OE-1, OE-2, OE-3, and OE-4. The data are presented as means values +/− s.d.

Supplementary Fig. S31. Predicting subcellular localization of TS using TargetP-2.0 software.

Supplementary Fig. S32 Subcellular localization analysis of TS without the 32 amino acids at the N-terminus. YFP, yellow fluorescence from YFP. MitoTracker Red, MitoTracker Red fluorescence-labelled mitochondria. Merge YFP+RED, the merged images for the yellow fluorescence and MitoTracker Red fluorescence. Chlorophyll, chlorophyll spontaneous fluorescence. Bright, bright field image. Overlapping images of all the channels mentioned above were merged. $TS^{Del\text{-}N32}$-YFP, TS without the 32 amino acids at the N-terminus fused with YFP. Tobacco transformation and microscopic analysis were independently conducted three times.

Supplementary Fig. S33 Effect of removing 32 amino acids at the N-terminus on the catalytic activity of TS. (A) The removal of 32 amino acids at the N-terminus resulted in an extreme decrease in catalytic activity. Recombinant protein obtained from three independent transformants of TS and $TS^{Del\text{-}N32}$ for activity test. ***P* < 0.0001. (B) The 32 N-terminal amino acids include a β-sheet structure consisting of the TS core scaffold. 32 Amino acids at the N-terminus were highlighted in red. Statistical analysis was performed according to the two-sided independent sample *t*-test.

Supplementary Fig. S34. Protein surface representation of TS. (A) Left side (showing the 3*β*-tropanol entry channel) of the acyl donor, 3*β*-tropanol, and TS ternary complex model. (B) Front of the tigloyl-CoA, 3*β*-tropanol, and TS ternary complex model. (C) Right side (showing the tigloyl-CoA entry channel) of the acyl donor, 3*β*-tropanol, and TS ternary complex model.

Supplementary Fig. S35. Consensus protein design improve the catalytic activity. (A) Consensus protein design for TS. (B) Hydrogen bonding network around the 3*β*-tropanol entry channel in TS. (C) Hydrogen bonding network around the 3*β*-tropanol entry channel in TS^{S40T}. (D) *K*cat/*K*m of 3*β*-

tropanol in synthesizing 3*β*-tigloyloxytropane. ***P* = 0.0082 (TS^{S40T}), ***P* = 0.0001 (TS^{F46I}), ***P* = 0.0024 ($TS^{S40T-F46I}$). (E) *K*cat/*K*m of tigloyl-CoA in synthesizing 3*β*-tigloyloxytropane. ***P* = 0.0097 (TS^{S40T}), ***P* = 0.0003 (TS^{F46I}), ***P* = 0.0003 ($TS^{S40T-F46I}$). (F) *K*cat/*K*m of 3*β*-tropanol in synthesizing 3*β*-acetoxytropane. ***P* = 0.0045 (TS^{S40T}), ***P* = 0.0014 (TS^{F46I}), ***P* = 0.0037 ($TS^{S40T-F46I}$). (G) *K*cat/*K*m of acetyl-CoA in synthesizing 3*β*-acetoxytropane. ***P* = 0.0016 (TS^{S40T}), ***P* = 0.0003 (TS^{F46I}), ***P* = 0.0005 ($TS^{S40T-F46I}$). (H) *K*cat/*K*m of 3*β*-tropanol in synthesizing 3*β*-benzoyloxytropane. ***P* = 0.0011 (TS^{S40T}), ***P* = 0.0002 (TS^{F46I}), ***P* = 0.0002 ($TS^{S40T-F46I}$). (I) *K*cat/*K*m of benzoyl-CoA in synthesizing 3*β*-benzoyloxytropane. **P* = 0.0107 (TS^{S40T}), ***P* = 0.0004 (TS^{F46I}), ***P* = 0.0007 ($TS^{S40T-F46I}$). Light gray dashed lines represent hydrogen bonds. Recombinant protein obtained from three independent transformants of TS and each mutant for activity test. The data are presented as means values +/− s.d. Statistical analysis was performed according to the two-sided independent sample *t*-test.

Supplementary Fig. S36 Enhancing the synthesis of tigloyl-CoA did not increase the yield of 3*β*-tigloyloxytropane in tobacco reconstructed via the biosynthetic pathway. The data are presented as means values +/− s.d. Leaves from three independent plants of each line was used for metabolite analysis.

Supplementary Fig. S37 Co-expressing PcICS and TS in tobacco and feeding 3*β*-tropanol and tiglic acid to produce 3*β*-tigloyloxypropane. A. LC–MS analysis of 3*β*-tigloyloxypropane in tobacco extracts. B. The contents of 3*β*-tigloyloxypropane in tobacco extracts. The data are presented as means values +/− s.d. Leaves from three independent plants of every line was used for metabolite analysis. Statistical analysis was performed according to the two-sided independent sample *t*-test. DW, dry weight.

Supplementary Table S1. Primers used in this study.

Name	Application	Primer sequence (5’→3‘’)
TS-BamHI-F	Construction of overexpression vector pBI121-TS	cgcggatccatggcctcagctgcattgaa
TS-SacI-R		cgcgagctcctaaaaataatttgcatatggag
TSvigs-XhoI-F	Construction of VIGS vector pTRVII-TS	cgcctcgagcctacaactactgaagtcct
TSvigs-KpnI-R		cgcggtaccgaaattcttcatcaccctcaa
TS-q-F	Quantitative Real-time PCR	ggattgccaccccagaagaa
TS-q-R		ggtcgtagccattggtttgc
35Spromoter-F	Plant genomic PCR detection for transgenic hairy root cultures	ctatccttcgcaagacccttc
TS-SacI-R		cgcgagctcctaaaaataatttgcatatggag
XhoI-TS-F	Construction of Subcellular localisation analysis vector	gcgctcgagatggcctcagctgcattgaaag
HindIII-TS-R		ggcaagcttaaaataatttgcatatggag
XhoI-32N-F		cgcctcgagatgcaaatatcaaaacttcttga
HindIII-32N-R		cgcaagcttaaatttatgccatttttgagtta
pMAL-BamHI-TSopt-F	Construction of MBP-tagged purification vectorp MAL-c5x-TS	tatcgtcgacggatccatggcaagcgcggc
pMAL-PstI-TSopt-R		gcttatttaattacctgcagttaaaaataatttgcatacgg
TS-AgeI-F	Construction of plant expression vector pEAQ-HT-TS	cgcaccggtatggcctcagctgcattgaa
TS-XhoI-R		cgcctcgagctaaaaataatttgcatatggag
AgeI-EnODC-F	Construction of plant expression vector pEAQ-HT-EnODC	cgcaccggtatgggttcgaacgccag
XhoI-EnODC-R		cgcctcgagctacggattggaataggc
AgeI-AbPMT-F	Construction of plant expression vector pEAQ-HT-AbPMT	cgcaccggtatggaggtcataagcaa
XhoI-AbPMT-R		cgcctcgagtcaaaactcaaccaaa
AgeI-AbPYKS-F	Construction of plant expression vector pEAQ-HT-AbPYKS	cgcaccggtatgaagttggaaaat
XhoI-AbPYKS-R		cgcctcgagttaaatgggcacactac
AgeI-AbCYP82M3-F	Construction of plant expression vector pEAQ-HT-AbCYP82M3	cgcaccggtatgtatgataattttctc
XhoI- AbCYP82M3-R		gcgctcgagctaaaattcataaagcacag
AgeI-DsTRII-F	Construction of plant expression vector pEAQ-HT-DsTRII	cgcaccggtatggctggaaggtggaatcttga
Xhol-DsTRII-R		cgcctcgagttaaaaaccacaattagccataagtcc
TSopt-SacI- F		cgcgagctcgatggcaagcgcggccctgaa
TSopt-HindIII-R		cgcaagcttaaaataatttgcatacggacttgc

PcICS-BglII-F	Construction of vector pET-Duet-BadA-TSopt/pET-Duet-PcICS-TSopt	cgcagatctcaatgcgtgattatgaacatgttgt
PcICS-XhoI-R		cgcctcgagacccagggcctgctgttttg
BadA-BglII-F		cgcagatctcaatgaacgcagcggctgtga
BadA-XhoI-R		cgcctcgagttaacccagtacaccttcacg
TS-H162A-F	Construction of Mutants of TS	catgtctgagtgctaaaattggagat
TS-H162A-R		atctccaattttagcactcagacatg
TS-I35A-F		aattcagcctggctgaacaggccca
TS-I35A-R		tgggcctgttcagccaggctgaatt
TS-Q39A-F		ttgaacaggccgcgagccacacatat
TS-Q39A-R		atatgtgtggctcgcggcctgttcaa
TS-Y280A-F		aactgattcaggctgccgatctg
TS-Y280A-R		cagatcggcagcctgaatcagtt
TS-N298A-F		aatagtgttggtgctgtgctgagtca
TS-N298A-R		tgactcagcacagcaccaacactatt
TS-L300A-F		tggtaatgtggcgagtcactttagc
TS-L300A-R		gctaaagtgactcgccacattacca
TS-W340A-F		aagaaaacgcagcggcactggaaat
TS-W340A-R		atttccagtgccgctgcgttttctt
TS-S40T-F		acaggcccagacccacacatat
TS-S40T-R		atatgtgtgggtctgggcctgt
TS-F46I-F		catatgttcctattggttttttt
TS-F46I-R		aaaaaaaccaataggaacatatg

Supplementary Table S2. Gradient profile of mobile phase for LC-MS analyses of 3*β*-acetoxytropane, hygrine, tropinone, 3*α*-tropanol and 3*β*-tropanol.

Time (min)	Mobile phase A (%)	Mobile phase B (%)
0	5	95
2	5	95
3	15	85
10	22	78
11	60	40
12	60	40
12.01	5	95
14	5	95

Mobile phase A = 10 mM ammonium formate + 0.1% formic acid in water. Mobile phase B = acetonitrile.

Supplementary Table S3. Gradient profile of mobile phase for LC-MS analyses of littorine, hyoscyamine, scopolamine, 3*β*-tigloyloxytropane, 3*β*-benzoyloxytropane, tigloyl norpseudotropine, tigloyl 1-hydroxynorpseudotropine and calystegine A3.

Time (min)	Mobile phase A (%)	Mobile phase B (%)
0	99	1
2	99	1
4.5	85	15
7	75	25
8.5	50	50
9	5	95
10	2	98
11	99	1
12	99	1

Mobile phase A = 0.1% formic acid in water. Mobile phase B = acetonitrile

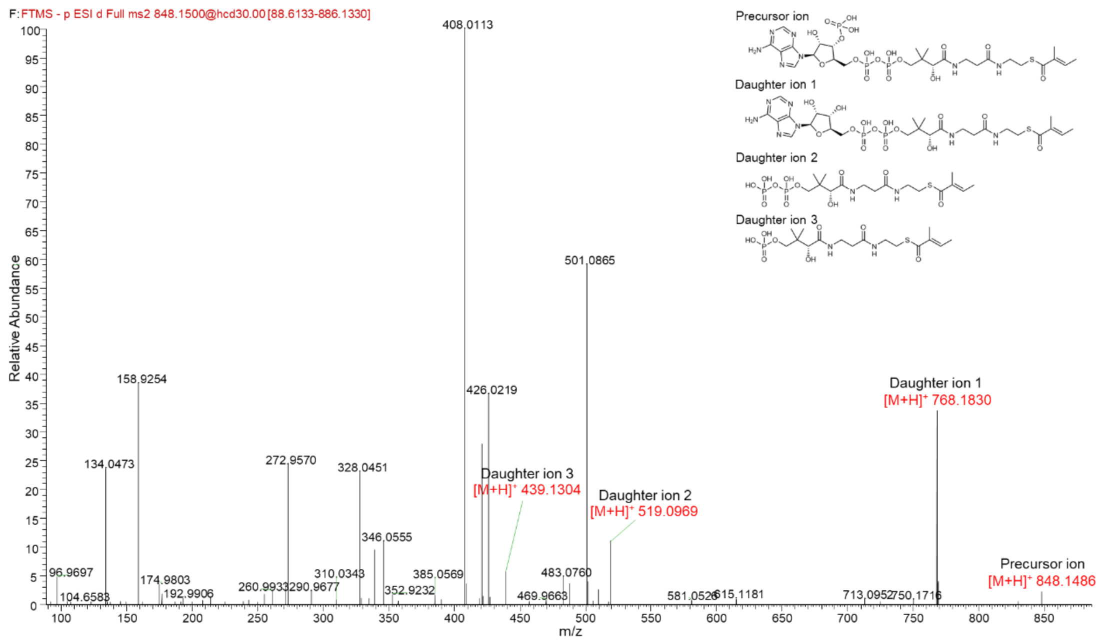

Supplementary Fig. S1. The MS/MS spectrum of Tigloyl-CoA.

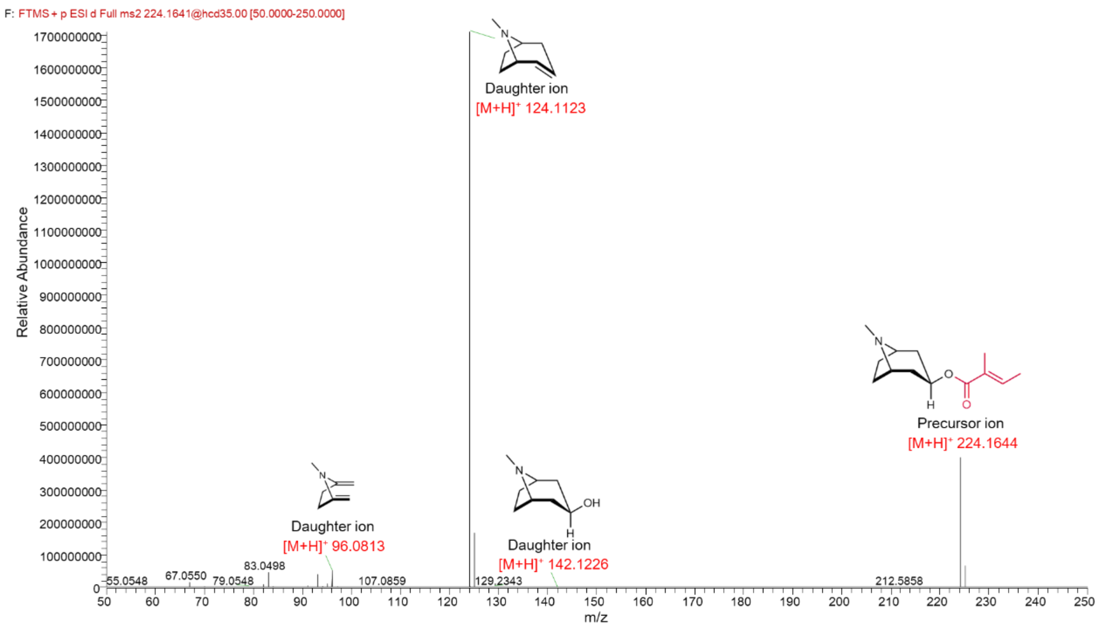

Supplementary Fig. S2. The MS/MS spectrum of 3*β*-tigloyloxytropane.

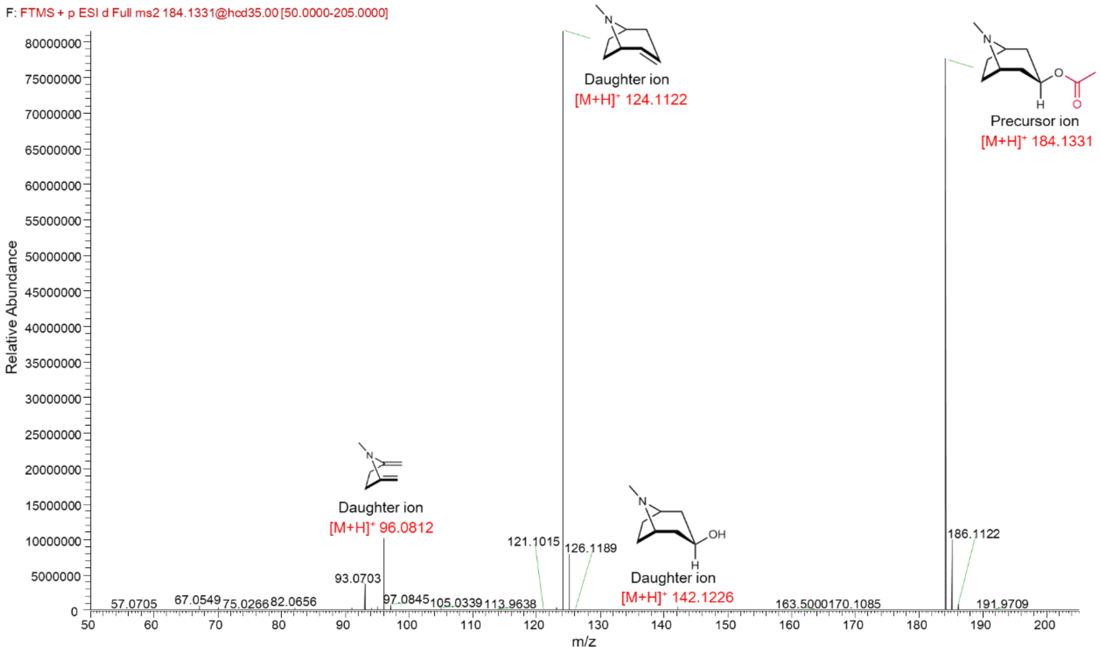

Supplementary Fig. S3. The MS/MS spectrum of 3*β*-acetoxytropane.

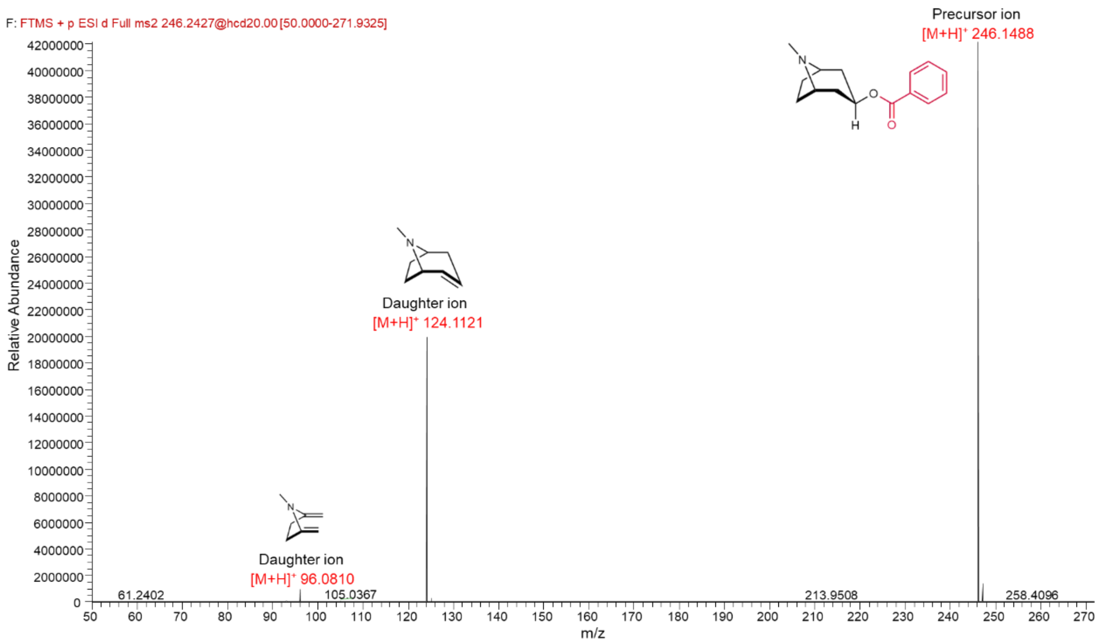

Supplementary Fig. S4. The MS/MS spectrum of 3*β*-benzoyloxytropane.

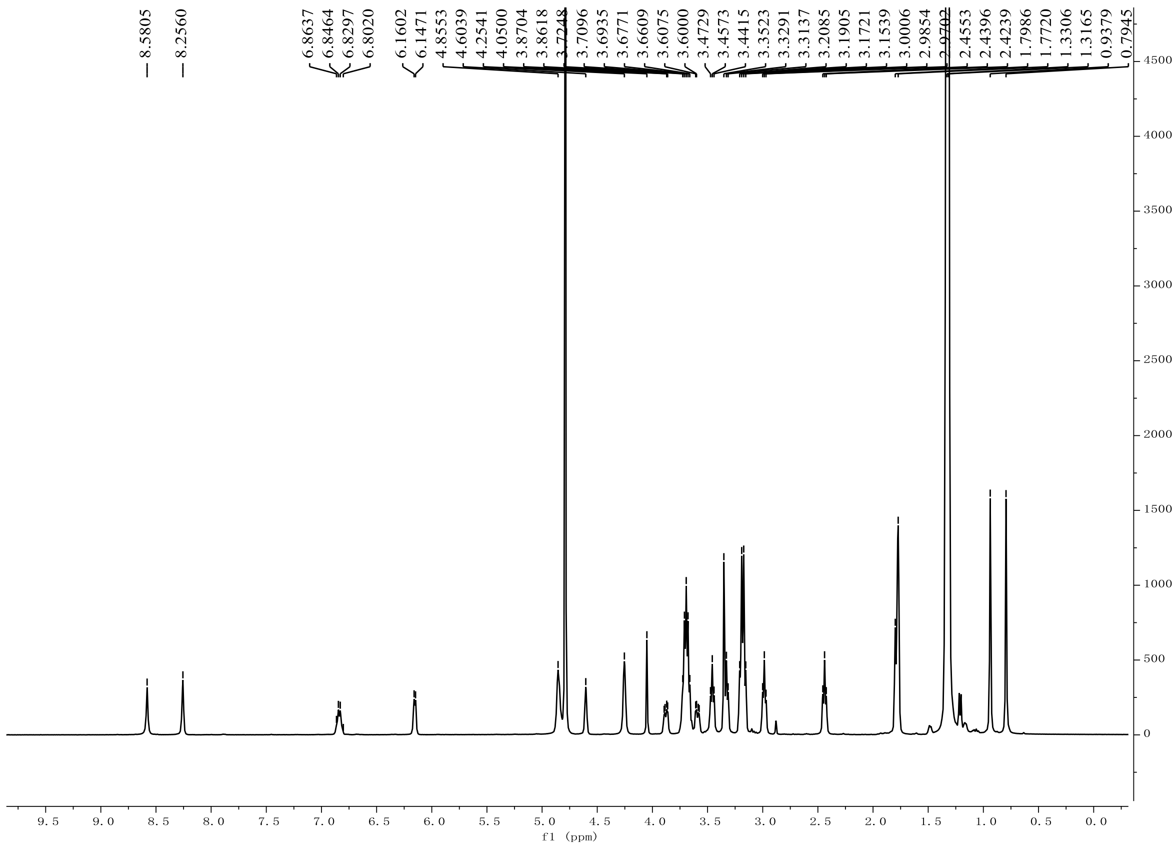

Supplementary Fig. S5. The ^{1}H–nuclear magnetic resonance (NMR) spectrum of tigloyl-CoA.

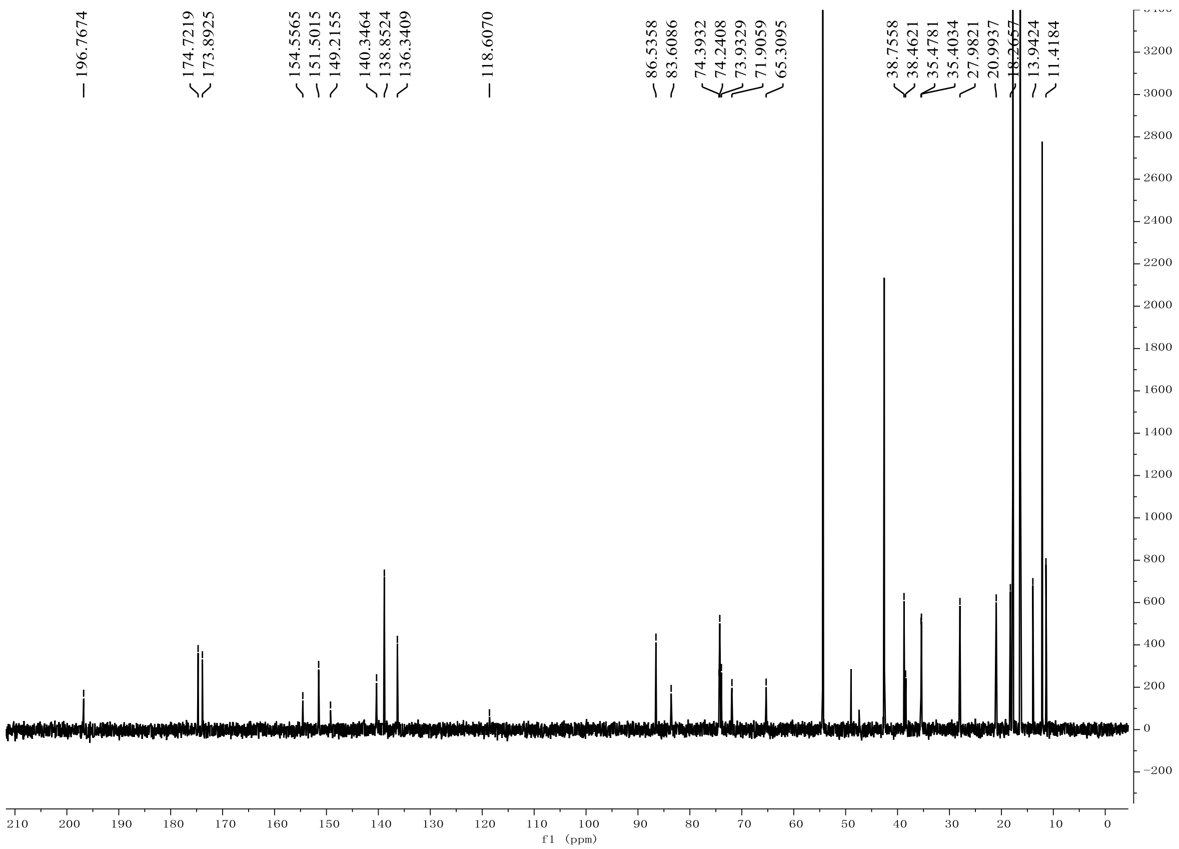

Supplementary Fig. S6. The ^{13}C-NMR spectrum of tigloyl-CoA.

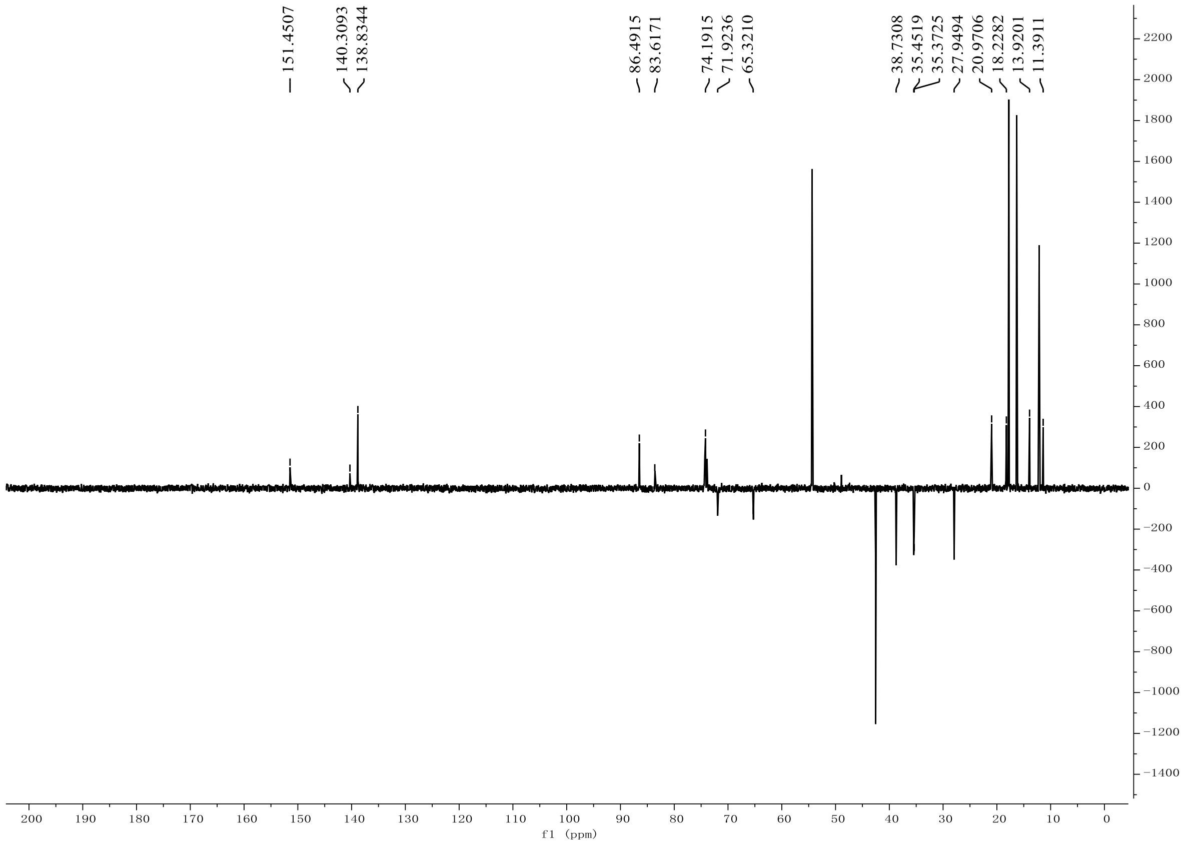

Supplementary Fig. S7. The distortionless enhancement by polarization transfer (DEPT) 135 NMR spectrum of tigloyl-CoA.

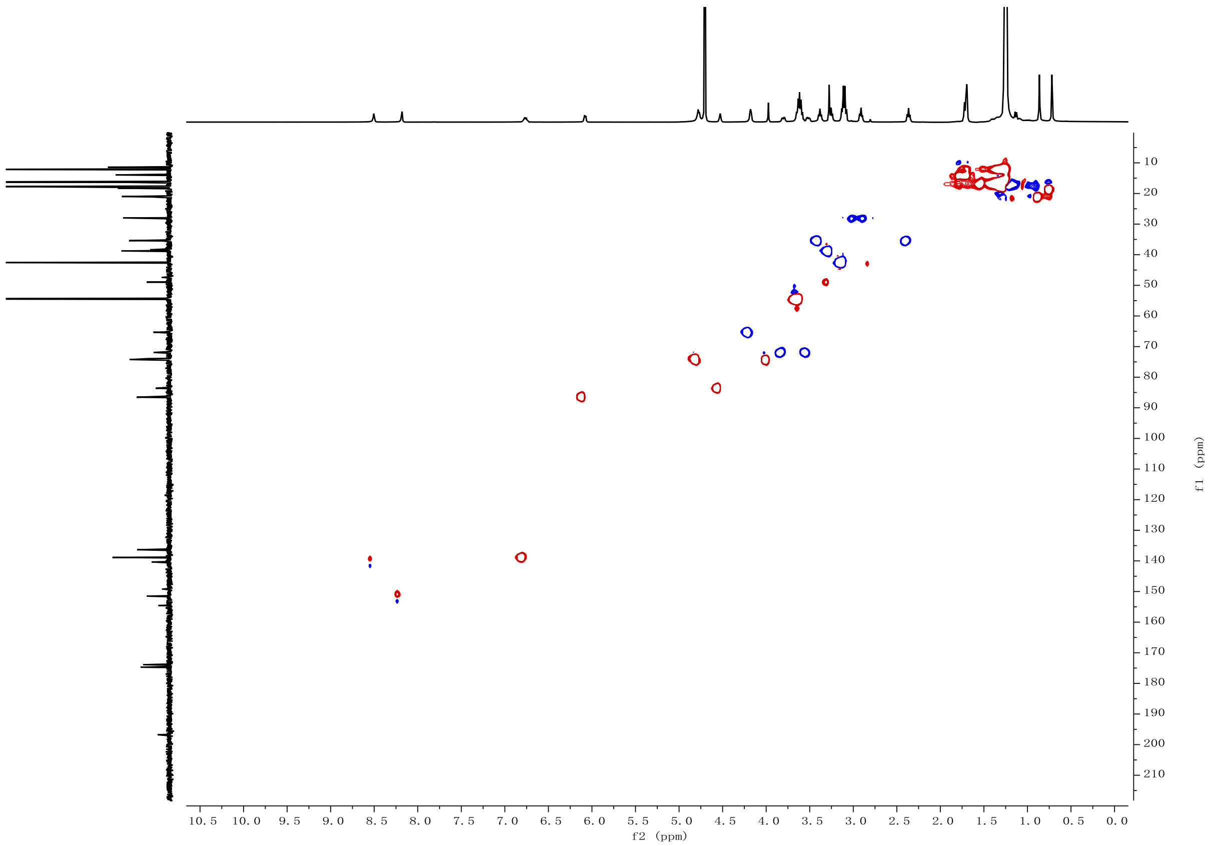

Supplementary Fig. S8. The heteronuclear single quantum coherence (HSQC) NMR spectrum of tigloyl-CoA.

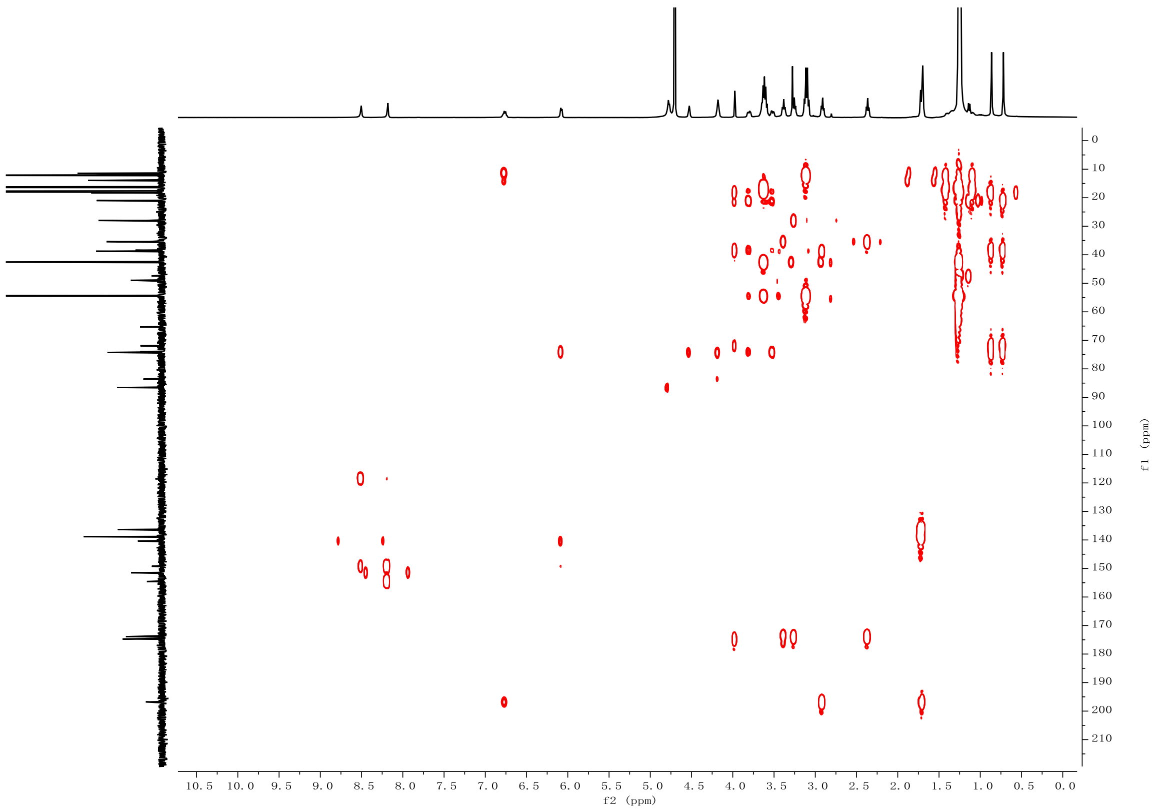

Supplementary Fig. S9. The heteronuclear multiple quantum coherence (HMBC) NMR spectrum of tigloyl-CoA.

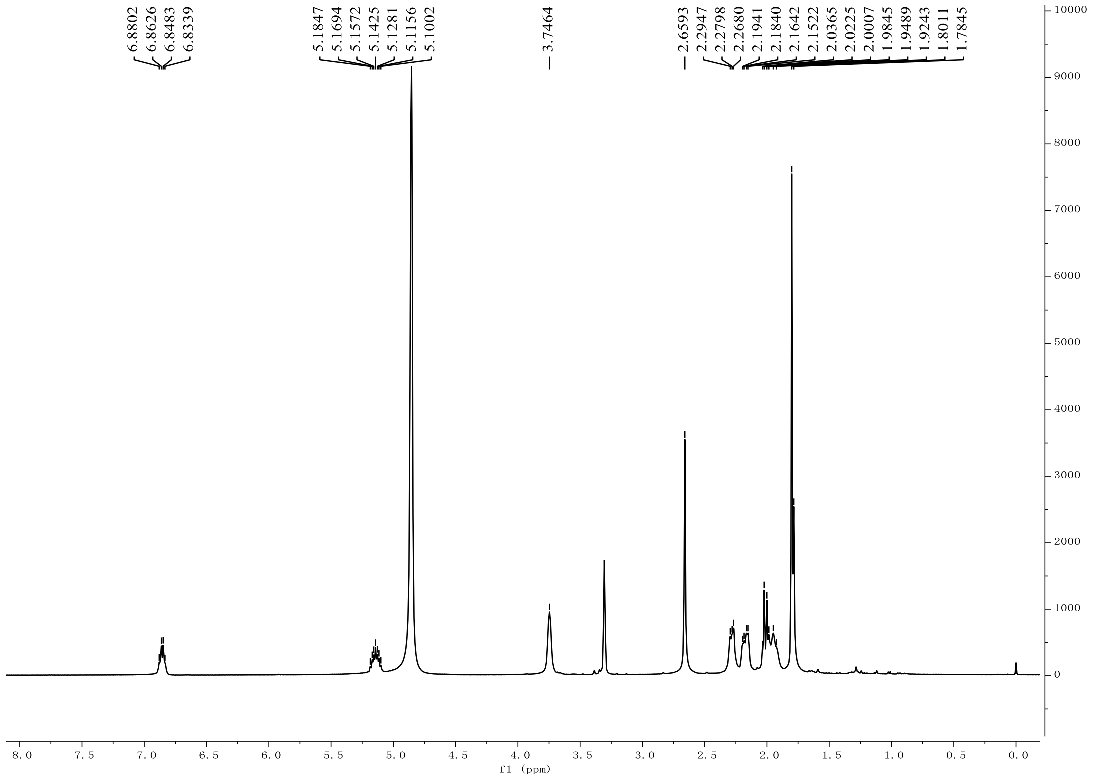

Supplementary Fig. S10. The ^{1}H–nuclear magnetic resonance (NMR) spectrum of 3*β*-tigloyloxytropane.

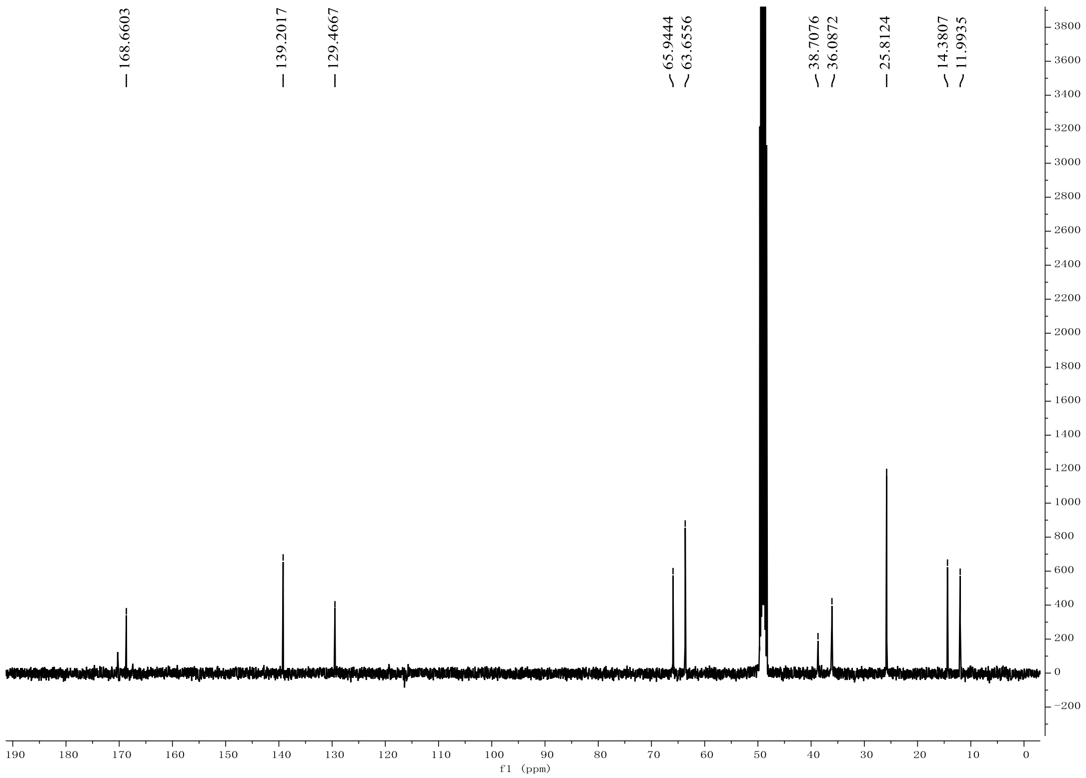

Supplementary Fig. S11. The ^{13}C-NMR spectrum of 3*β*-tigloyloxytropane.

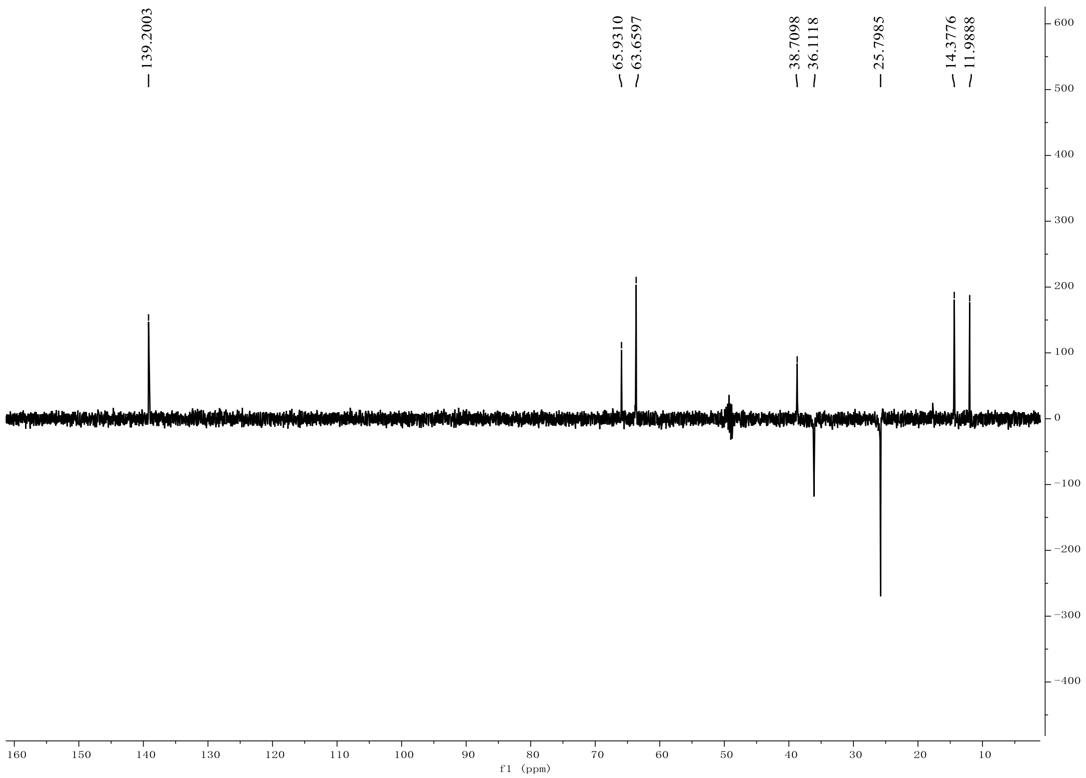

Supplementary Fig. S12. The distortionless enhancement by polarization transfer (DEPT) 135 NMR spectrum of 3*β*-tigloyloxytropane.

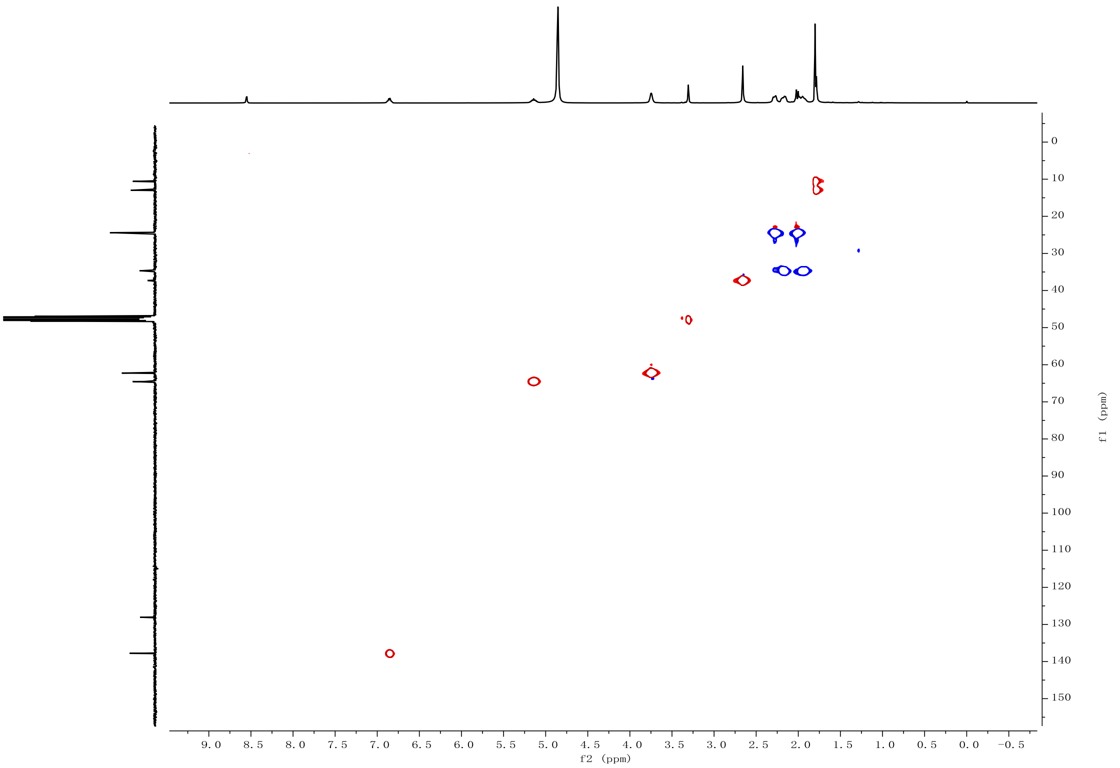

Supplementary Fig. S13. The heteronuclear single quantum coherence (HSQC) NMR spectrum of 3*β*-Tigloyloxytropane.

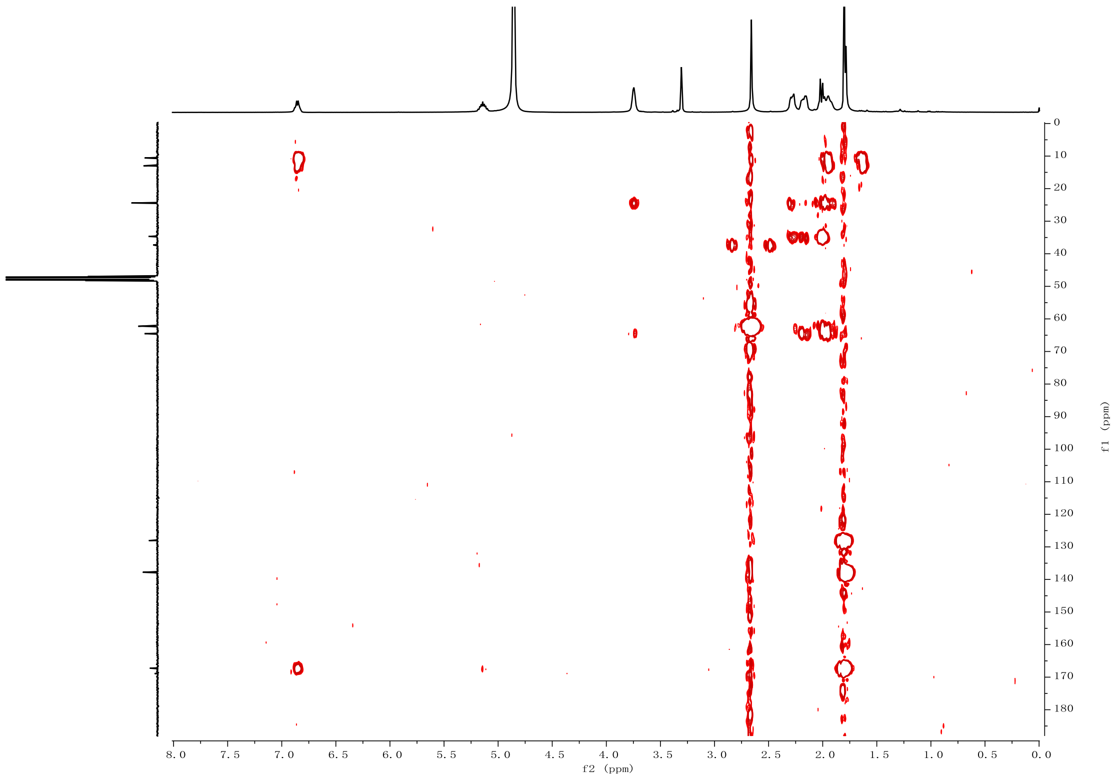

Supplementary Fig. S14. The heteronuclear multiple quantum coherence (HMBC) NMR spectrum of 3*β*-Tigloyloxytropane.

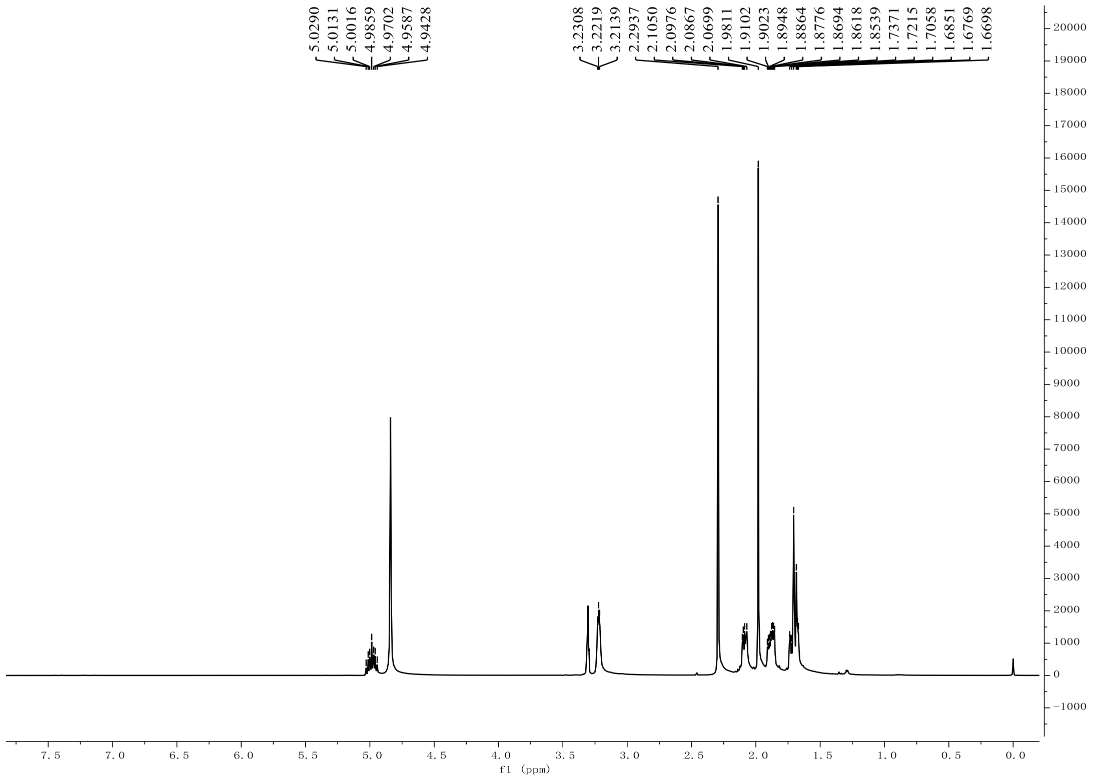

Supplementary Fig. S15. The ^{1}H-NMR spectrum of 3*β*-acetoxytropane.

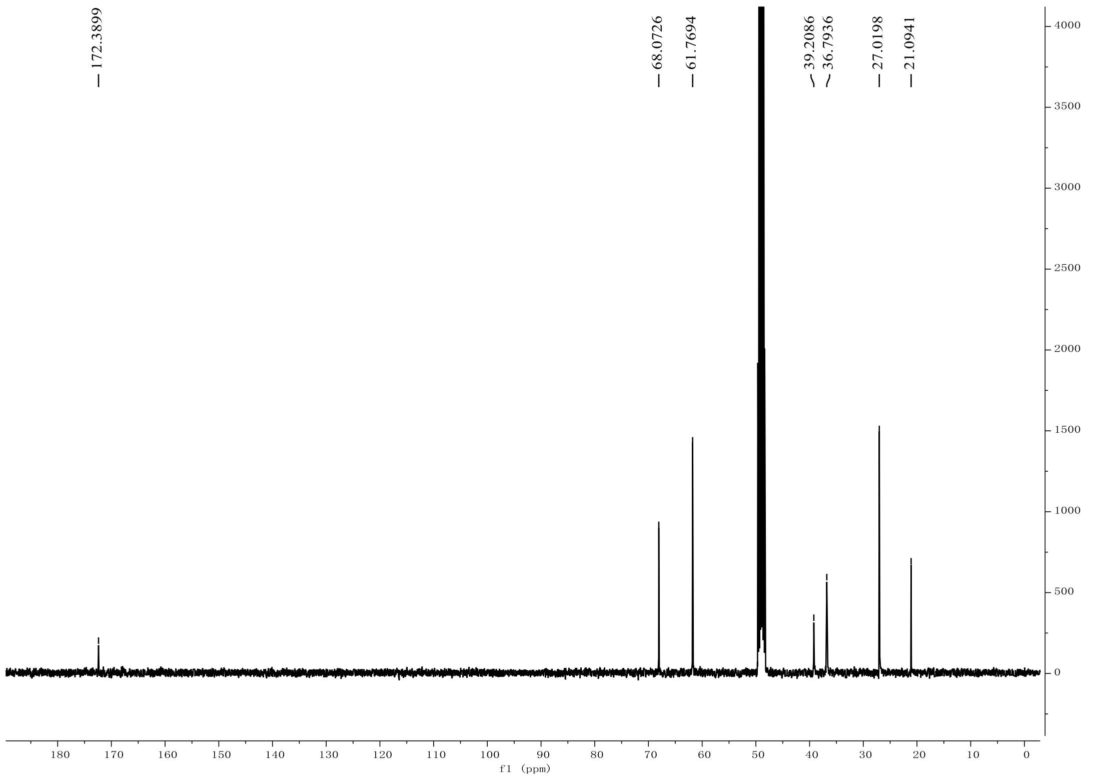

Supplementary Fig. S16. The ^{13}C-NMR spectrum of 3*β*-acetoxytropane.

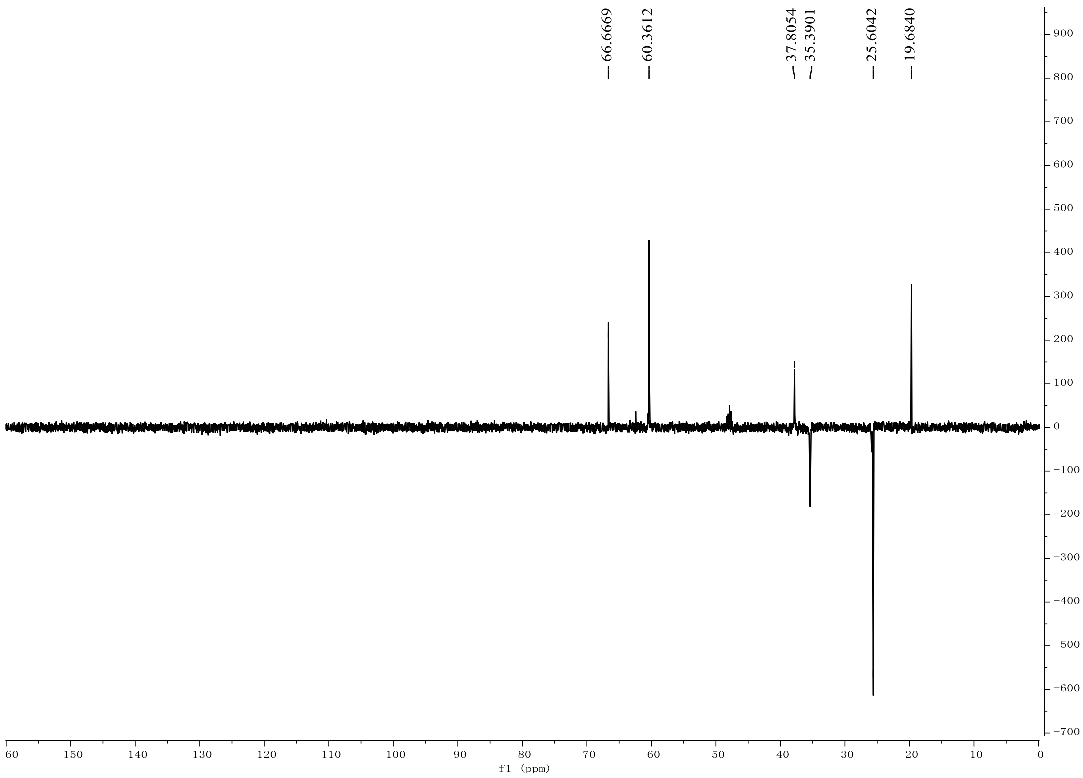

Supplementary Fig. S17. The DEPT 135 NMR spectrum of 3*β*-acetoxytropane.

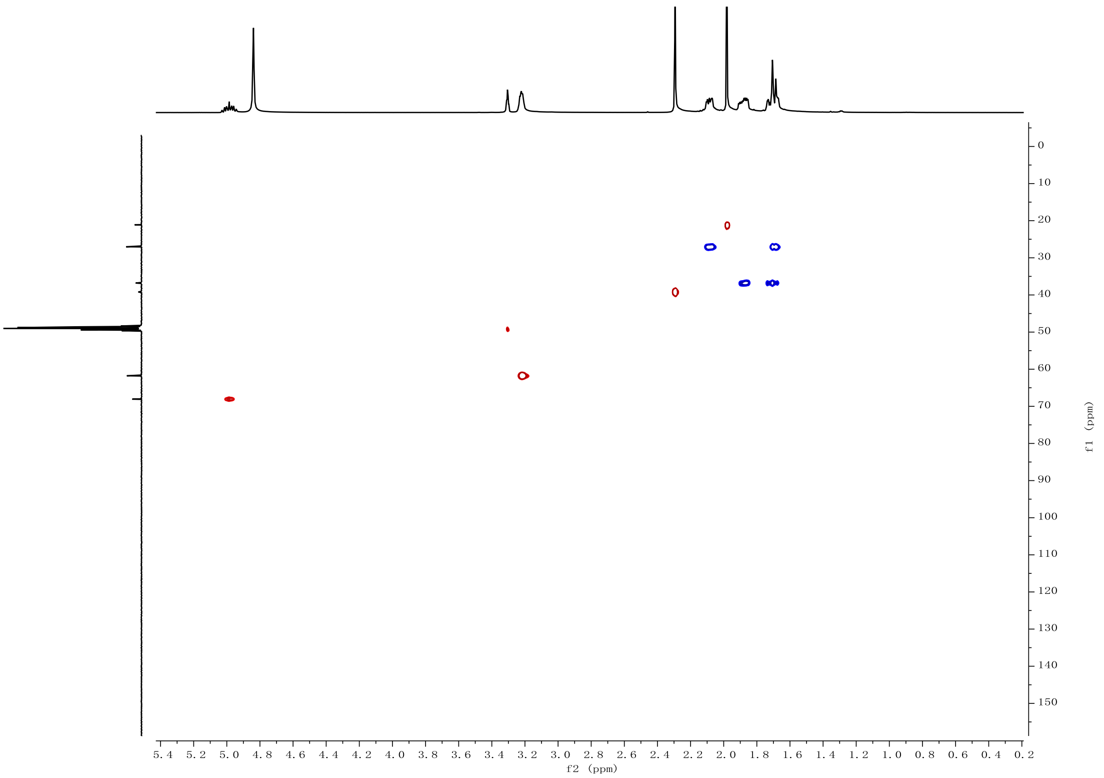

Supplementary Fig. S18. The HSQC NMR spectrum of 3*β*-acetoxytropane.

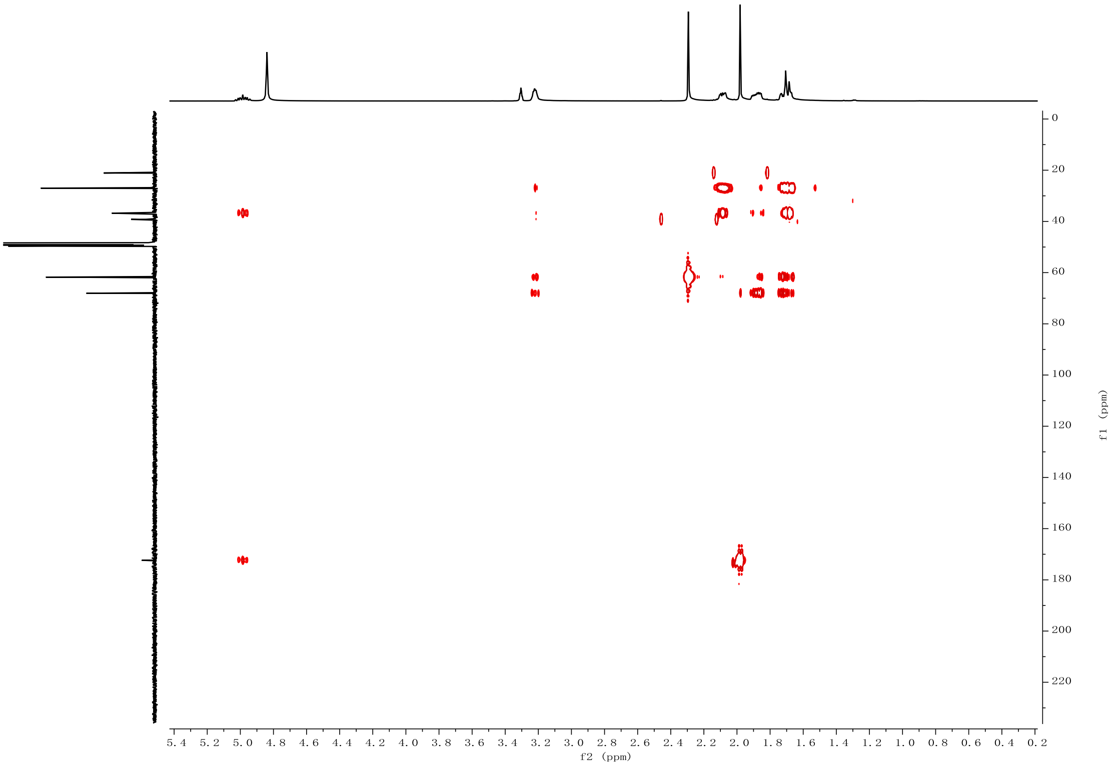

Supplementary Fig. S19. The HMBC NMR spectrum of 3*β*-acetoxytropane.

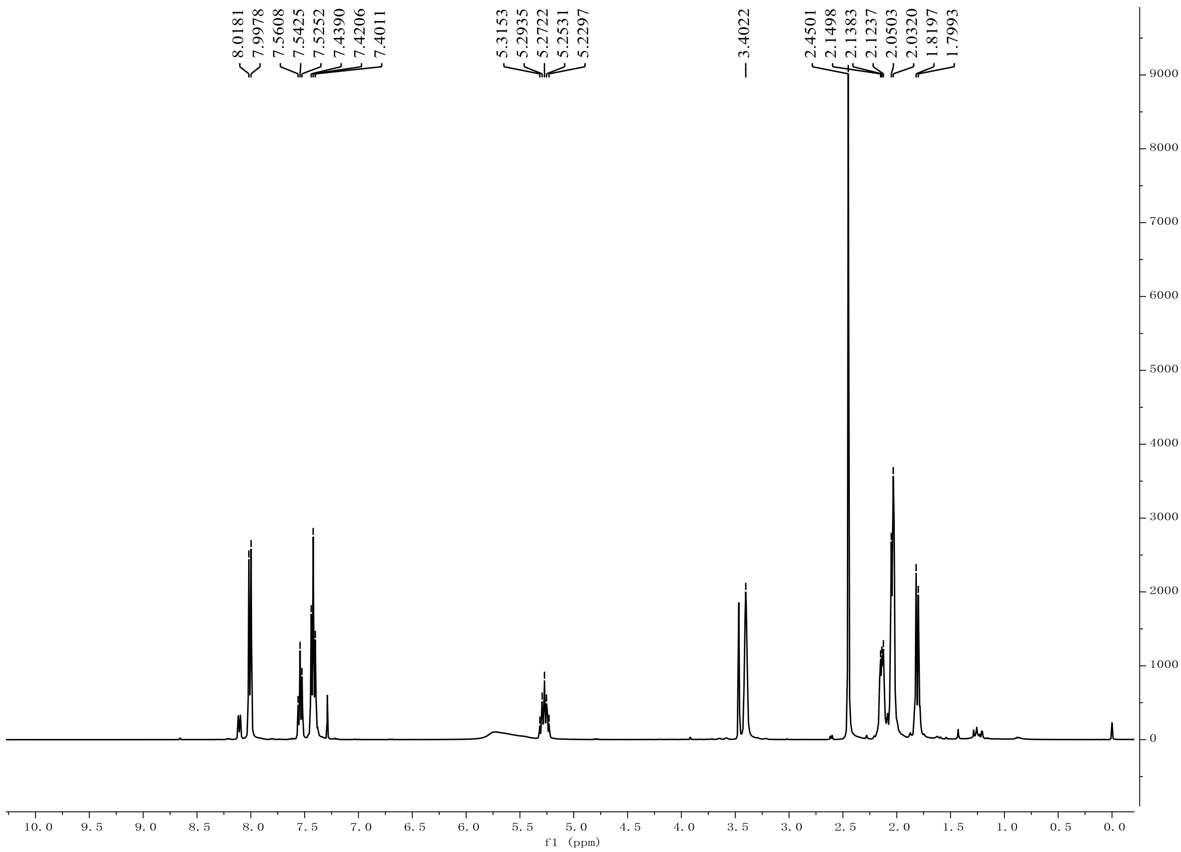

Supplementary Fig. S20. The ^{1}H-NMR spectrum of 3*β*-benzoyloxytropane.

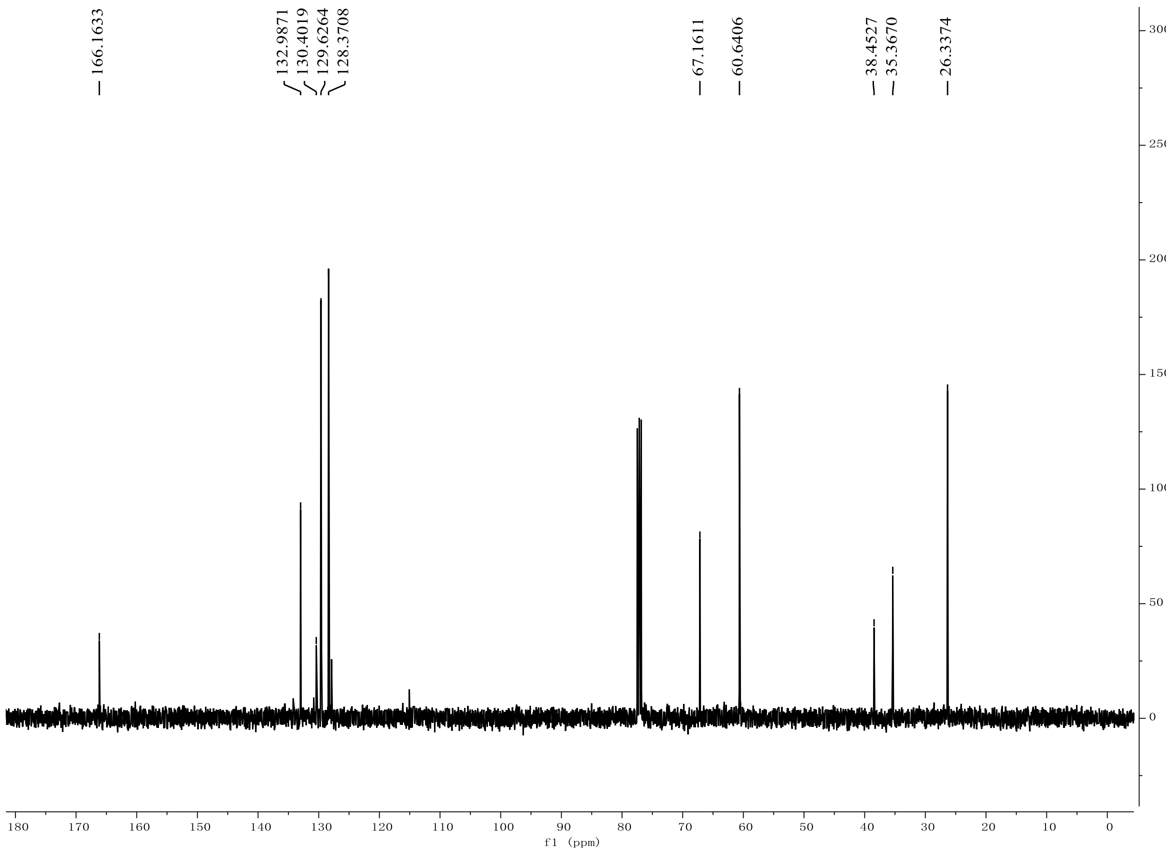

Supplementary Fig. S21. The ^{13}C-NMR spectrum of 3*β*-benzoyloxytropane.

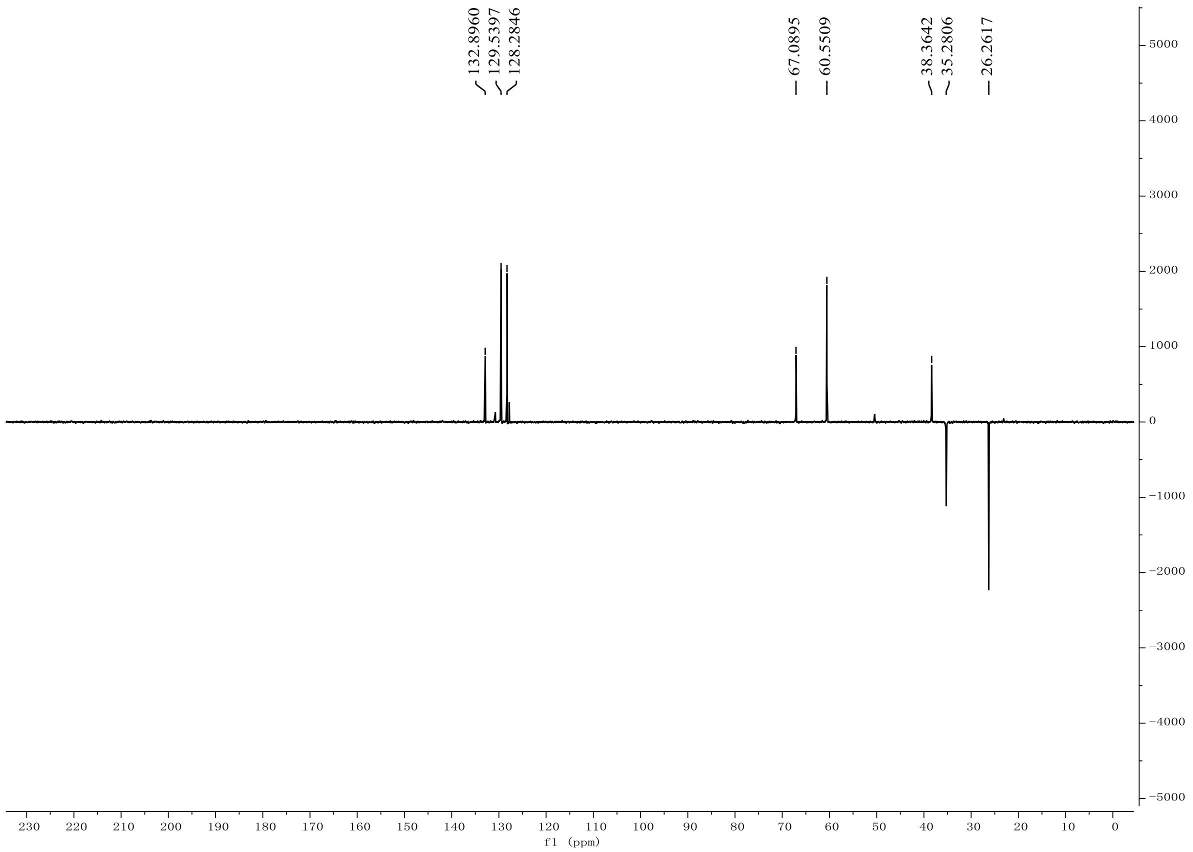

Supplementary Fig. S22. The DEPT 135 NMR spectrum of 3*β*-benzoyloxytropane.

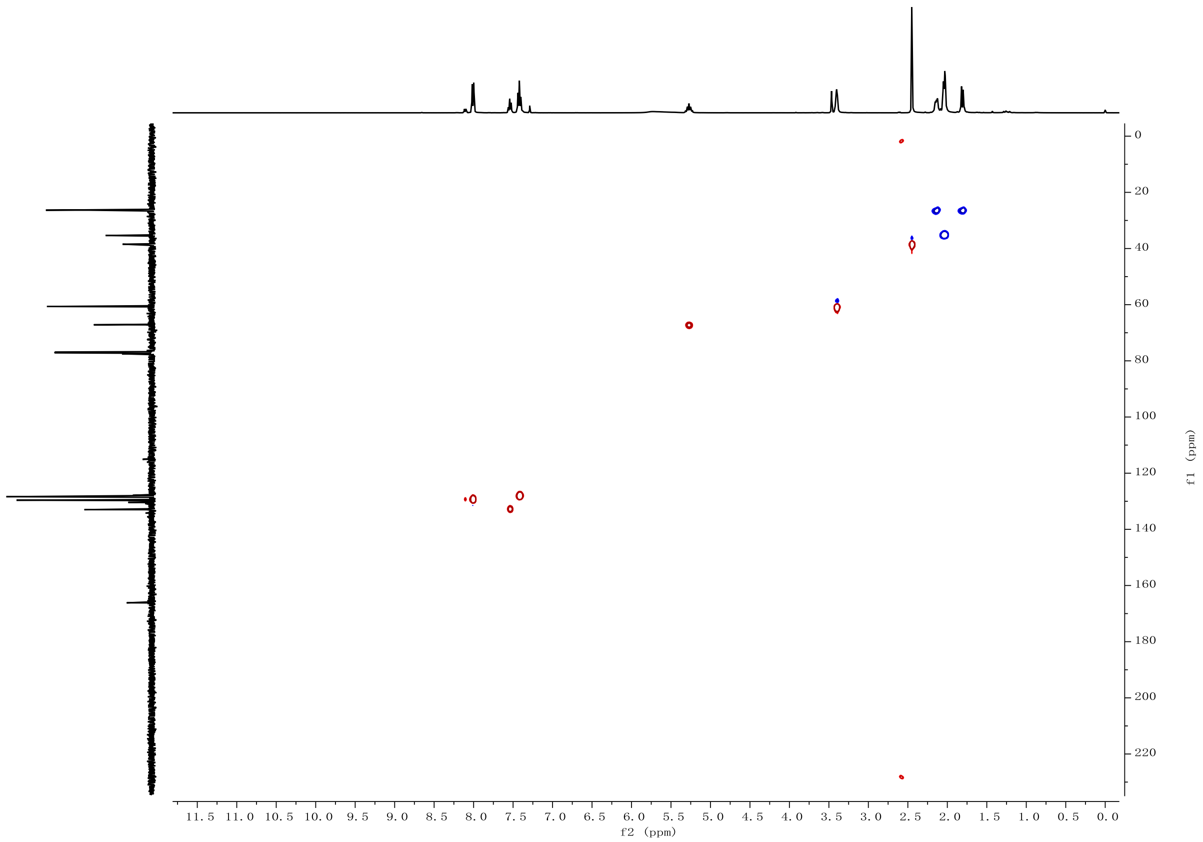

Supplementary Fig. S23. The HSQC NMR spectrum of 3*β*-benzoyloxytropane.

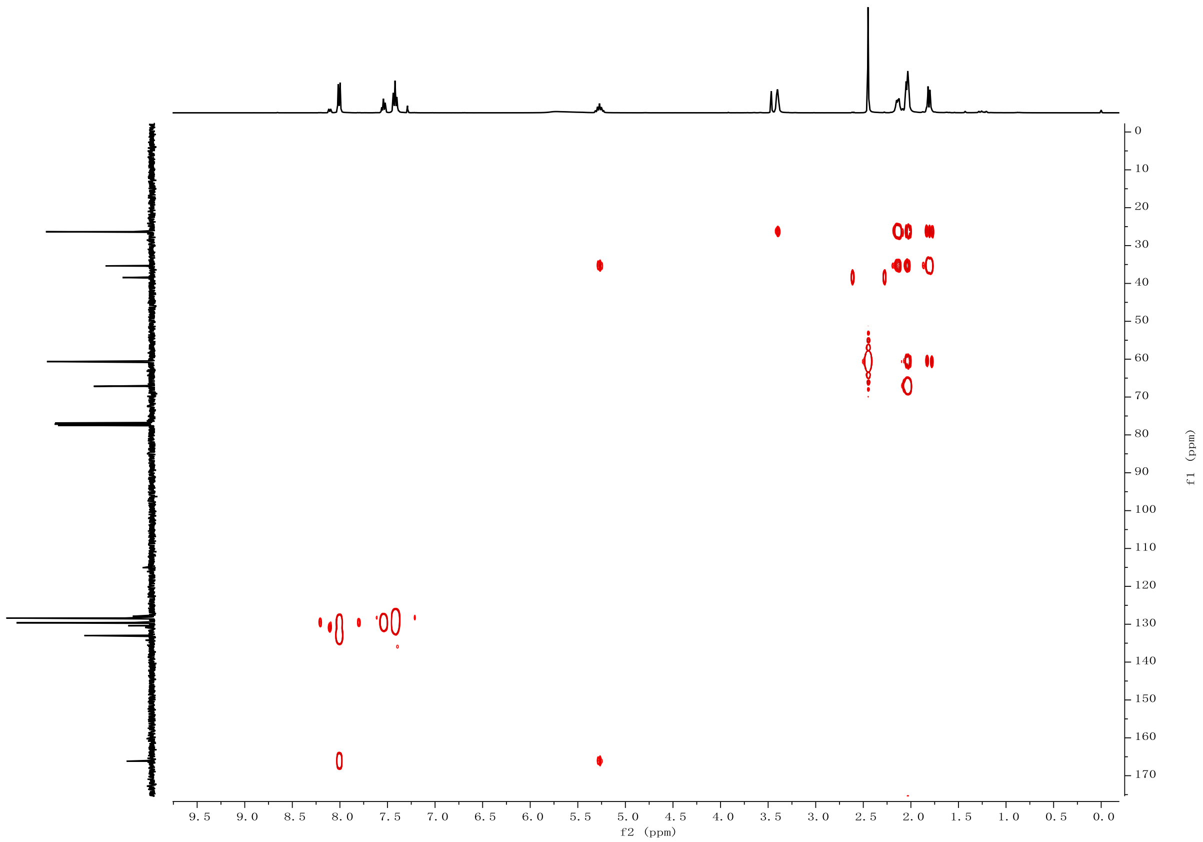

Supplementary Fig. S24. The HMBC NMR spectrum of 3*β*-benzoyloxytropane.

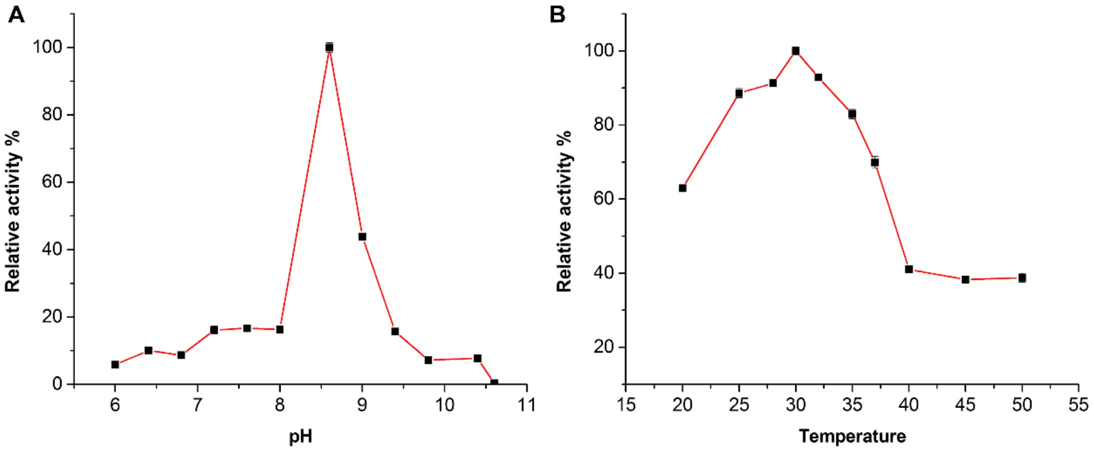

Supplementary Fig. S25. The analysis of optimum pH and temperature of TS. (A) The optimum pH analysis. (B) The optimum temperature analysis. Recombinant protein obtained from three independent transformants of TS for activity test. The data are presented as means values +/− s.d.

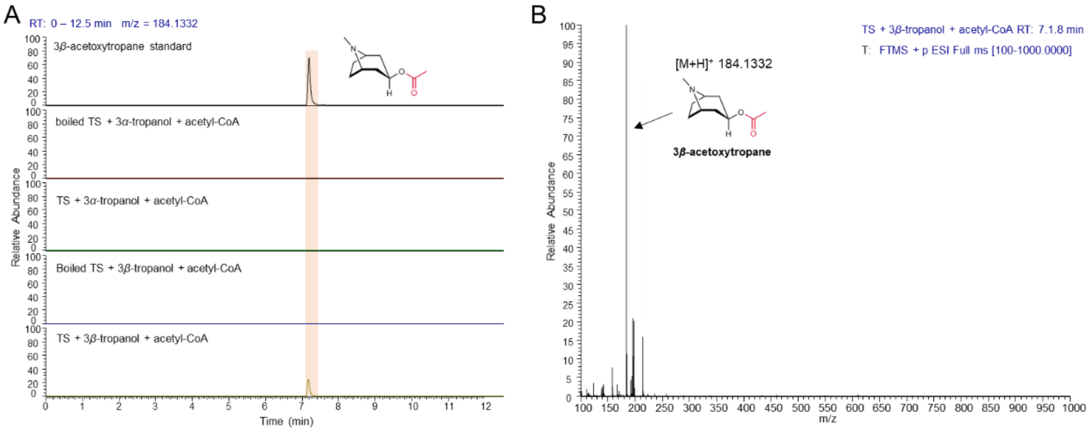

Supplementary Fig. S26. TS catalyses the condensation between 3*β*-tropanol and acetyl-CoA to generate 3*β*-acetoxytropane. (A) TS enzymatic assays with acetyl-CoA as acyl donor. (B) Mass spectrometry data of 3*β*-acetoxytropane.

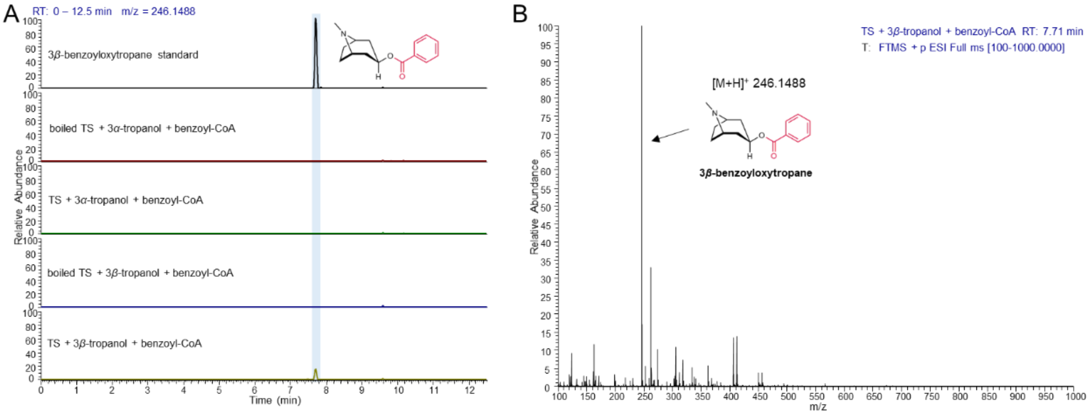

Supplementary Fig. S27. TS catalyses the condensation between 3*β*-tropanol and benzoyl-CoA to generate 3*β*-benzoyloxytropane. (A) TS enzymatic assays with benzoyl-CoA as acyl donor. (B) Mass spectrometry data of 3*β*-benzoyloxytropane.

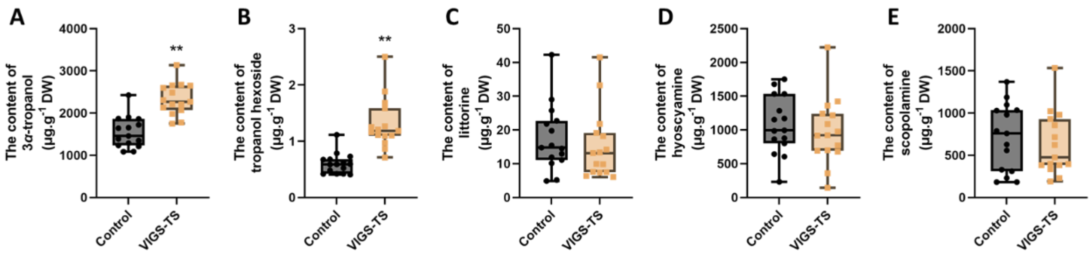

Supplementary Fig. S28. The influence of silencing TS on the content of compounds involved in the competitive metabolic flow of TS in *A. belladonna* seedlings. (A) 3α-Tropanol. ***P* < 0.0001. (B) Tropanol hexoside. ***P* < 0.0001. (C) Littorine. (D) Hyoscyamine. (E) Scopolamine. Fifteen independent plants were used in the VIGS assays. Center line of box plot denotes the median value; lower and upper bounds of box plot denote first and third quartile; whiskers of box plot extend to the smallest and maximum values. Statistical analysis was performed according to the two-sided independent sample *t*-test. DW, dry weight.

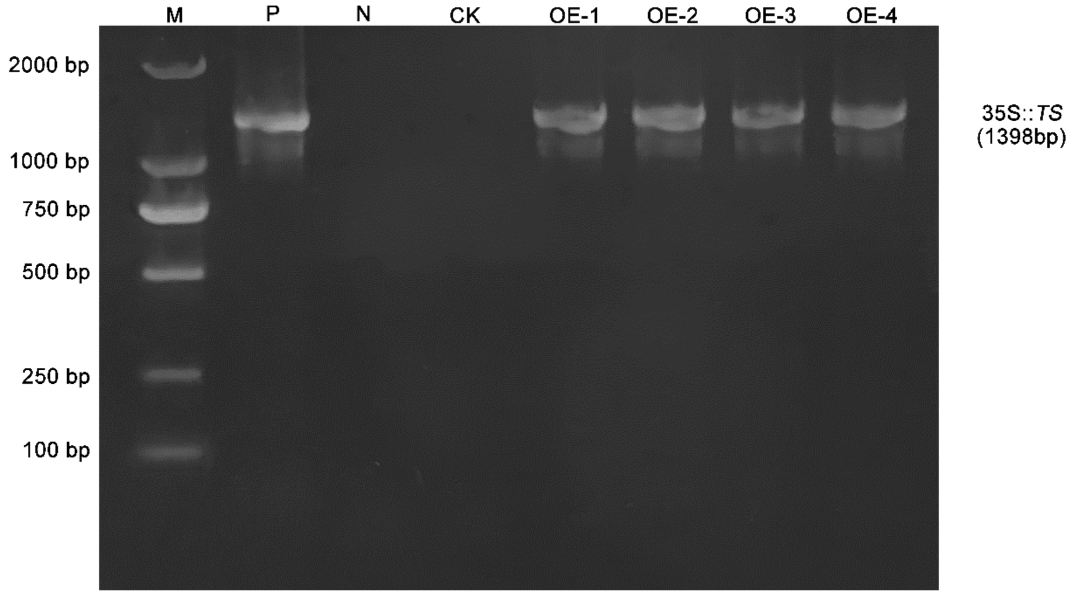

Supplementary Fig. S29. Plant genomic PCR detection for transgenic hairy root cultures. M, DNA marker. P, plasmid pBI121-TS, used as positive control. N, plasmid pBI121, used as negative control. CK, root cultures lines transformed by pBI121. TS, all independently transformed root cultures lines with overexpression of TS (three biological replicates for each line), including OE-1, OE-2, OE-3, and OE-4.

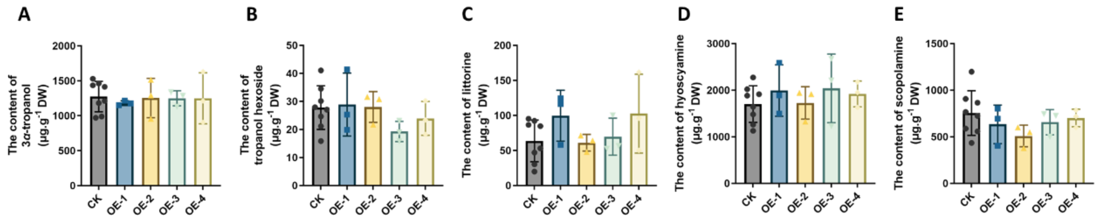

Supplementary Fig. S30. The influence of TS overexpression on the content of compounds in the competitive metabolic flow of TS in *A. belladonna* hairy root cultures. (A) 3*α*-Tropanol. (B) Tropanol hexoside. (C) Littorine. (D) Hyoscyamine. (E) Scopolamine. OE denotes all independently transformed root culture lines overexpressing TS (three biological replicates for each line), including OE-1, OE-2, OE-3, and OE-4. The data are presented as means values +/− s.d.

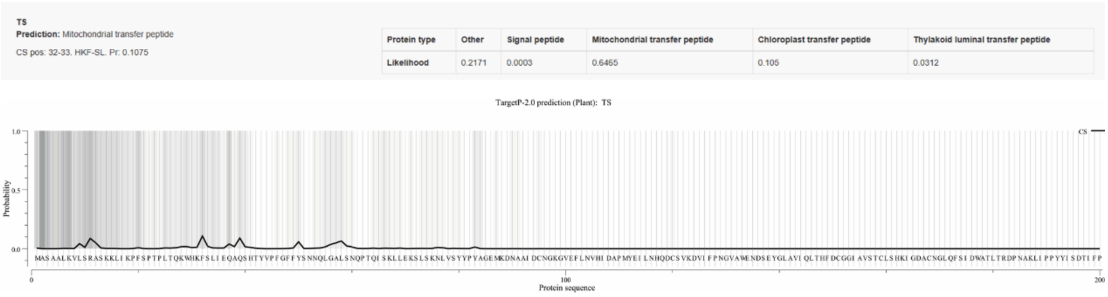

Supplementary Fig. S31. Predicting subcellular localization of TS using TargetP-2.0 software.

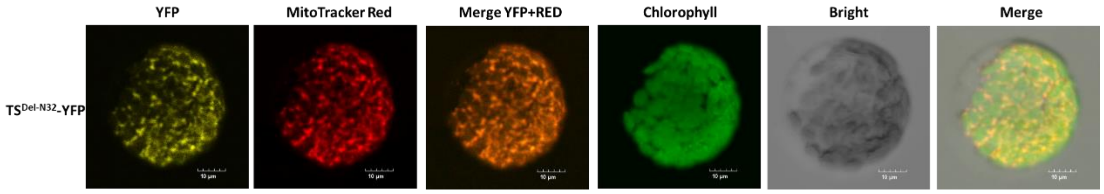

Supplementary Fig. S32 Subcellular localization analysis of TS without the 32 amino acids at the N-terminus. YFP, yellow fluorescence from YFP. MitoTracker Red, MitoTracker Red fluorescence-labelled mitochondria. Merge YFP+RED, the merged images for the yellow fluorescence and MitoTracker Red fluorescence. Chlorophyll, chlorophyll spontaneous fluorescence. Bright, bright field image. Overlapping images of all the channels mentioned above were merged. TS$^{Del-N32}$-YFP, TS without the 32 amino acids at the N-terminus fused with YFP. Tobacco transformation and microscopic analysis were independently conducted three times.

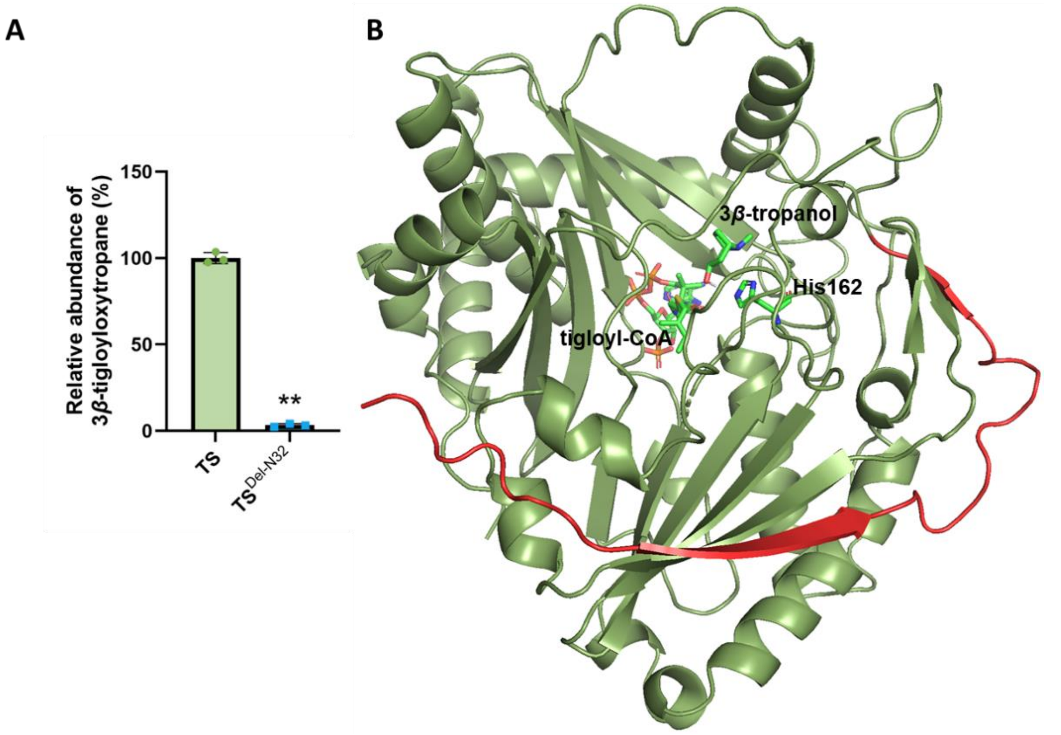

Supplementary Fig. S33 Effect of removing 32 amino acids at the N-terminus on the catalytic activity of TS. (A) The removal of 32 amino acids at the N-terminus resulted in an extreme decrease in catalytic activity. Recombinant protein obtained from three independent transformants of TS and $TS^{Del-N32}$ for activity test. **P < 0.0001. (B) The 32 N-terminal amino acids include a β-sheet structure consisting of the TS core scaffold. 32 Amino acids at the N-terminus were highlighted in red. Statistical analysis was performed according to the two-sided independent sample *t*-test.

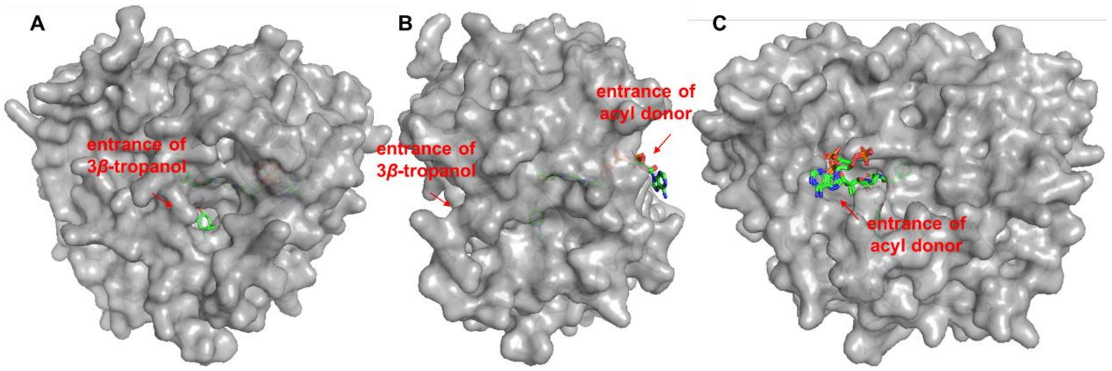

Supplementary Fig. S34. Protein surface representation of TS. (A) Left side (showing the 3*β*-tropanol entry channel) of the acyl donor, 3*β*-tropanol, and TS ternary complex model. (B) Front of the tigloyl-CoA, 3*β*-tropanol, and TS ternary complex model. (C) Right side (showing the tigloyl-CoA entry channel) of the acyl donor, 3*β*-tropanol, and TS ternary complex model.

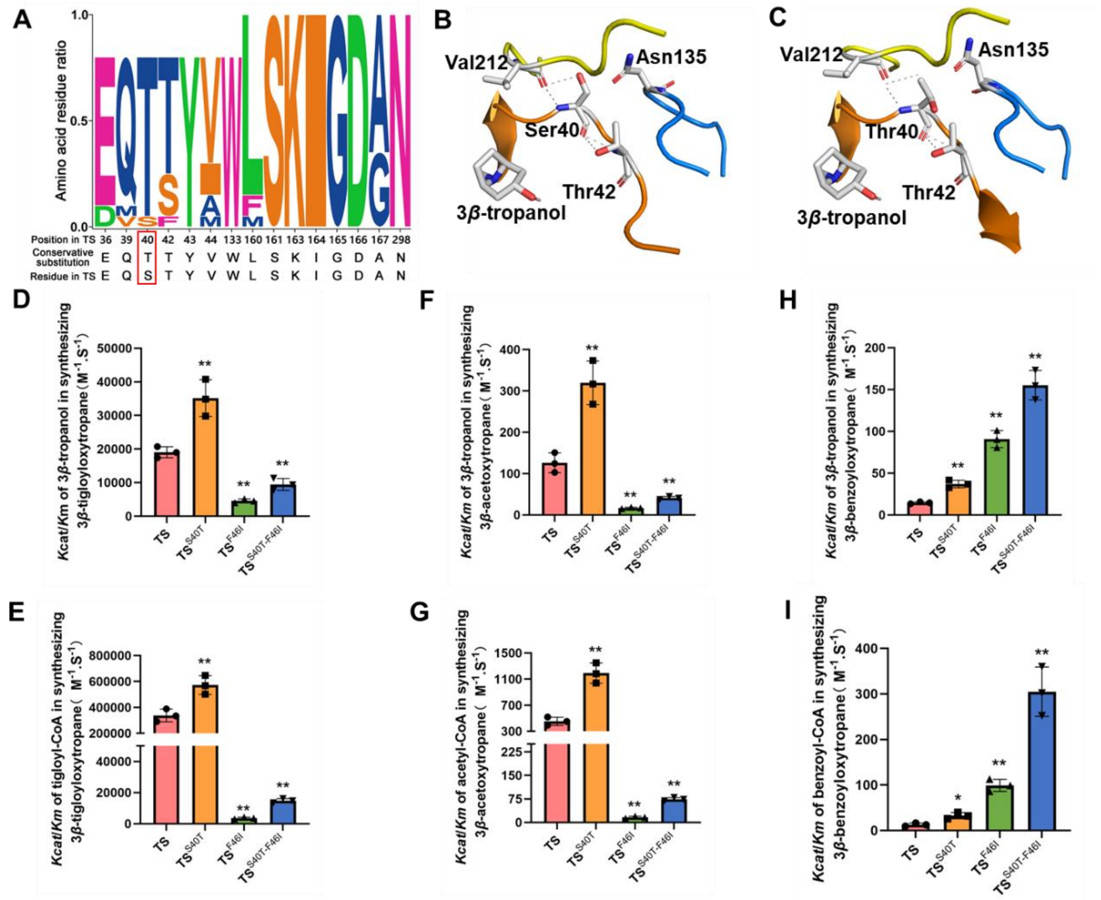

Supplementary Fig. S35. Consensus protein design improve the catalytic activity. (A) Consensus protein design for TS. (B) Hydrogen bonding network around the 3*β*-tropanol entry channel in TS. (C) Hydrogen bonding network around the 3*β*-tropanol entry channel in TS^{S40T}. (D) *K*cat/*K*m of 3*β*-tropanol in synthesizing 3*β*-tigloyloxytropane. $**P = 0.0082$ (TS^{S40T}), $**P = 0.0001$ (TS^{F46I}), $**P = 0.0024$ ($TS^{S40T\text{-}F46I}$). (E) *K*cat/*K*m of tigloyl-CoA in synthesizing 3*β*-tigloyloxytropane. $**P = 0.0097$ (TS^{S40T}), $**P = 0.0003$ (TS^{F46I}), $**P = 0.0003$ ($TS^{S40T\text{-}F46I}$). (F) *K*cat/*K*m of 3*β*-tropanol in synthesizing 3*β*-acetoxytropane. $**P = 0.0045$ (TS^{S40T}), $**P = 0.0014$ (TS^{F46I}), $**P = 0.0037$ ($TS^{S40T\text{-}F46I}$). (G) *K*cat/*K*m of acetyl-CoA in synthesizing 3*β*-acetoxytropane. $**P = 0.0016$ (TS^{S40T}), $**P = 0.0003$ (TS^{F46I}), $**P = 0.0005$ ($TS^{S40T\text{-}F46I}$). (H) *K*cat/*K*m of 3*β*-tropanol in synthesizing 3*β*-benzoyloxytropane. $**P = 0.0011$ (TS^{S40T}), $**P = 0.0002$ (TS^{F46I}), $**P = 0.0002$ ($TS^{S40T\text{-}F46I}$). (I) *K*cat/*K*m of benzoyl-CoA in synthesizing 3*β*-benzoyloxytropane. $*P = 0.0107$ (TS^{S40T}), $**P = 0.0004$ (TS^{F46I}), $**P = 0.0007$ ($TS^{S40T\text{-}F46I}$). Light gray dashed lines represent hydrogen bonds. Recombinant protein obtained from three independent transformants of TS and each mutant for activity test. The data are presented as means values +/− s.d. Statistical analysis was performed according to the two-sided independent sample *t*-test.

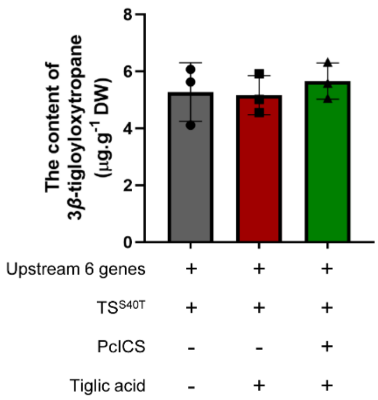

Supplementary Fig. S36 Enhancing the synthesis of tigloyl-CoA did not increase the yield of 3*β*-tigloyloxytropane in tobacco reconstructed via the biosynthetic pathway. The data are presented as means values +/− s.d. Leaves from three independent plants of each line was used for metabolite analysis.

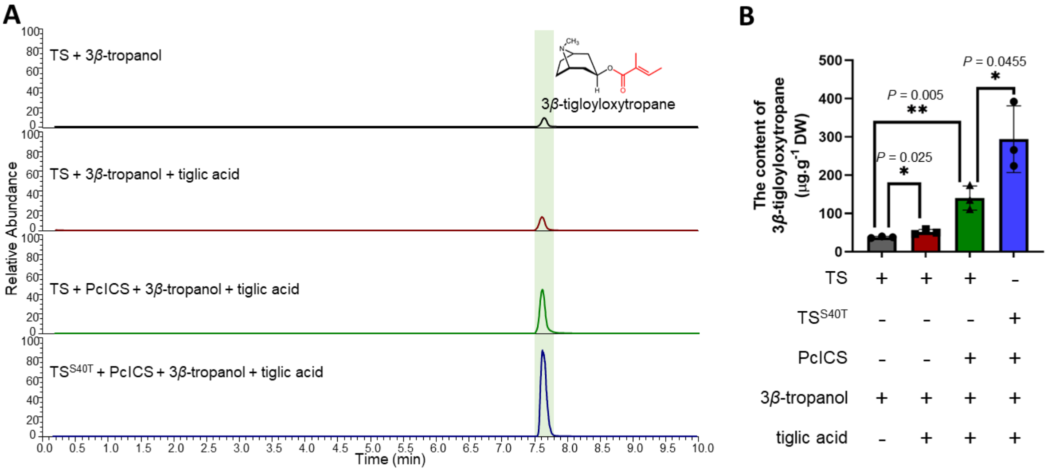

Supplementary Fig. S37 Co-expressing PcICS and TS in tobacco and feeding 3*β*-tropanol and tiglic acid to produce 3*β*-tigloyloxypropane. A. LC–MS analysis of 3*β*-tigloyloxypropane in tobacco extracts. B. The contents of 3*β*-tigloyloxypropane in tobacco extracts. The data are presented as means values +/− s.d. Leaves from three independent plants of every line was used for metabolite analysis. Statistical analysis was performed according to the two-sided independent sample *t*-test. DW, dry weight.
