# Supplementary material for: Discovering a mitochondrion-localized BAHD acyltransferase involved in calystegine biosynthesis and engineering the production of 3β-tigloyloxytropane
Source: Nat Commun. 2024 Apr 29;15:3623. doi: 10.1038/s41467-024-47968-0 (PMC11058270; doi:10.1038/s41467-024-47968-0)
Supplement: Supplementary file 3 — Description of Additional Supplementary Files [file 41467_2024_47968_MOESM3_ESM.pdf]

Description of Additional Supplementary Files

Supplementary Data Legends:

Supplementary Data 1. The sequences (46 BAHD acyltransferases of *Atropa belladonna* and 196 functionally identified BAHD acyltransferases) used to construct the phylogenetic tree.

Supplementary Data 2. Phylogenetic analysis of BAHD acyltransferases.

Supplementary Data 3. The ^{1}H-NMR, ^{13}C-NMR, and 2D-NMR data of the chemicals synthesized in this study.
