# Supplementary figures and images for: Discovering a mitochondrion-localized BAHD acyltransferase involved in calystegine biosynthesis and engineering the production of 3β-tigloyloxytropane
Source: Nat Commun. 2024 Apr 29;15:3623. doi: 10.1038/s41467-024-47968-0 (PMC11058270; doi:10.1038/s41467-024-47968-0)

bootstrap

- 46
- 59.5
- 73
- 86.5
- 100

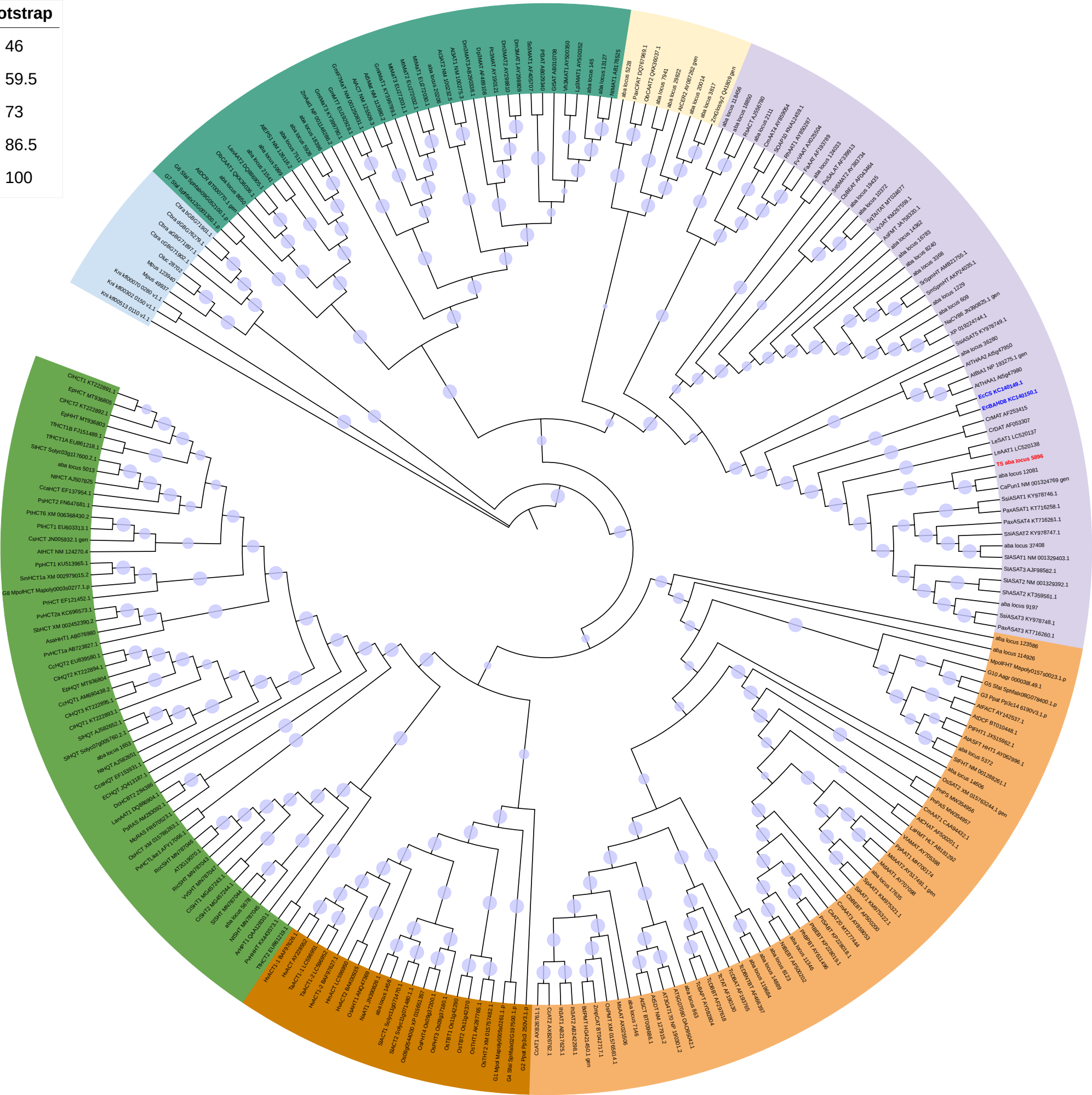

Supplement: Supplementary file 5 — Supplementary Data 2 [file 41467_2024_47968_MOESM5_ESM.pdf]
