# Supplementary material for: Discovering a mitochondrion-localized BAHD acyltransferase involved in calystegine biosynthesis and engineering the production of 3β-tigloyloxytropane
Source: Nat Commun. 2024 Apr 29;15:3623. doi: 10.1038/s41467-024-47968-0 (PMC11058270; doi:10.1038/s41467-024-47968-0)
Supplement: Supplementary file 6 — Supplementary Data 3 [file 41467_2024_47968_MOESM6_ESM.docx]

NMR data for chemicals is as follows:

Tigloyl-CoA: ^1^H NMR (400 MHz, CDCl_3_) *δ*_H_: 8.58 (1H, s, H-1''), 8.26 (1H, s, H-4''), 6.84 (1H, q, *J* = 6.68 Hz, H-2), 6.15 (1H, d, *J* = 5.25 Hz, H-1'), 4.86 (2H, m, H-H-2', 4'), 4.60 (1H, br s, H-3'), 4.25 (2H, br s, H-5''), 4.05 (1H, s, H-11), 3.88 (1H, dd, *J* = 9.31, 3.41 Hz, H-13a), 3.59 (1H, dd, *J* = 9.41, 3.11 Hz, H-13b), 3.46 (2H, t, *J* = 6.30 Hz, H-9), 3.32 (2H, t, *J* = 6.14 Hz, H-6), 2.99 (2H, t, *J* = 6.09 Hz, H-5), 2.44 (2H, t, *J* = 6.28 Hz, H-8), 1.79 (3H, d, *J* = 6.68 Hz, H-1), 1.77 (3H, s, H-14), 0.94 (3H, s, H-15), 0.79 (3H, s, H-16); ^13^C NMR (100 MHz, CDCl_3_) *δ*_C_: 196.8 (C-4), 174.7 (C-10), 173.9 (C-7), 154.6 (C-3''), 151.5 (C-4''), 149.2 (C-5''), 140.3 (C-1''), 138.9 (C-2), 136.3 (C-3), 118.6 (C-2''), 86.5 (C-1'), 83.6 (C-3'), 74.4 (C-4'), 74.2 (C-11), 73.9 (C-2'), 71.9 (C-13), 65.3 (C-5'), 38.8 (C-6), 38.5 (C-12), 38.4, 35.5 (C-8), 35.4 (C-9), 28.0 (C-5), 21.1 (C-15), 21.0 (), 18.3 (C-16), 13.9 (C-1), 11.4 (C-14).

3*β*-Acetoxytropane: ^1^H-NMR (CD_3_OD, 400 MHz) *δ*_H_: 4.99 (1H, ddd, *J* = 18.23, 10.97, 6.34 Hz, H-1), 3.22 (2H, m, H-3, 6), 2.29 (3H, s, -NCH_3_), 2.09 (2H, dd, *J* = 9.21, 4.84 Hz, H-4a, 5a), 1.98 (3H, s, 2′-CH_3_), 1.88 (2H, ddd, 13.04, 6.19, 3.17 Hz, H-2a, 7a), 1.71 (2H, m, H-2b, 7b), 1.67 (2H, m, H-4b, 5b); ^13^C-NMR (CD_3_OD, 100 MHz) *δ*_C_: 172.4 (C-1′), 68.1 (C-1), 61.8 (C-3, 6), 39.2 (N-CH_3_), 36.8 (C-2, 7), 27.0 (C-4, 5), 21.1 (C-2′).

3*β*-Tigloyloxytropane: ^1^H-NMR (CD_3_OD, 400 MHz) *δ*_H_: 6.86 (1H, q, *J* = 6.40 Hz, H-4′), 5.14 (1H, tt, *J* = 11.17, 6.16 Hz, H-1), 3.75 (2H, br s, H-3, 6), 2.66 (3H, s, N-CH_3_), 2.28 (2H, m, H-4a, 5a), 2.17 (2H, dd, *J* = 12.36, 4.33 Hz, H-2a, 7a), 2.01 (2H, m, H-4b, 5b), 1.95 (2H, m, H-2b, 7b), 1.80 (3H, s, 5′-CH_3_), 1.79 (3H, d, *J* = 6.64 Hz, 4′-CH_3_); ^13^C-NMR (CD_3_OD, 100 MHz) *δ*_C_: 168.7 (C-1′), 139.2 (C-3′), 129.5 (C-2′), 65.9 (C-1), 63.7 (C-3), 38.7 (N-CH_3_), 36.1 (C-2, 7), 25.8 (C-4, 5), 14.4 (C-4′), 12.0 (C-5′).

3*β*-Benzoyloxytropane: ^1^H NMR (400 MHz, CDCl_3_) *δ*_H_: 8.01 (2H, d, *J* = 8.12 Hz, H-2', 6'), 7.54 (1H, t, *J* = 7.13 Hz, H-4'), 7.42 (2H, t, *J* = 7.58 Hz, H-3', 5'), 5.27 (1H, m, H-1), 3.40 (2H, m, H-3, 6), 2.45 (3H, s, N-CH_3_), 2.18-2.02 (6H, m, H-2, 7, 4a, 5a), 1.81 (2H, d, *J* = 8.16 Hz, H-4b, 5b); ^13^C NMR (100 MHz, CDCl_3_) *δ*_C_: 166.2 (C-7'), 133.0 (C-4'), 130.4 (C-1'), 129.6 (C-2', 6'), 128.4 (C-3', 5'), 67.2 (C-1), 60.6 (C-3, 6), 38.5 (N-CH_3_), 35.4 (C-2, 7), 26.3 (C-4, 5).
