# Supplementary material for: Discovering a mitochondrion-localized BAHD acyltransferase involved in calystegine biosynthesis and engineering the production of 3β-tigloyloxytropane
Source: Nat Commun. 2024 Apr 29;15:3623. doi: 10.1038/s41467-024-47968-0 (PMC11058270; doi:10.1038/s41467-024-47968-0)
Supplement: Supplementary file 7 — Reporting Summary [file 41467_2024_47968_MOESM7_ESM.pdf]

nature portfolio

Corresponding author(s): Zhihua Liao

Last updated by author(s): Apr 1, 2024

Reporting Summary

Nature Portfolio wishes to improve the reproducibility of the work that we publish. This form provides structure for consistency and transparency in reporting. For further information on Nature Portfolio policies, see our Editorial Policies and the Editorial Policy Checklist.

Statistics

For all statistical analyses, confirm that the following items are present in the figure legend, table legend, main text, or Methods section.

n/a	Confirmed	
☐	☒	The exact sample size (*n*) for each experimental group/condition, given as a discrete number and unit of measurement
☐	☒	A statement on whether measurements were taken from distinct samples or whether the same sample was measured repeatedly
☐	☒	The statistical test(s) used AND whether they are one- or two-sided<br>*Only common tests should be described solely by name; describe more complex techniques in the Methods section.*
☒	☐	A description of all covariates tested
☐	☒	A description of any assumptions or corrections, such as tests of normality and adjustment for multiple comparisons
☐	☒	A full description of the statistical parameters including central tendency (e.g. means) or other basic estimates (e.g. regression coefficient) AND variation (e.g. standard deviation) or associated estimates of uncertainty (e.g. confidence intervals)
☐	☒	For null hypothesis testing, the test statistic (e.g. *F*, *t*, *r*) with confidence intervals, effect sizes, degrees of freedom and *P* value noted<br>*Give P values as exact values whenever suitable.*
☒	☐	For Bayesian analysis, information on the choice of priors and Markov chain Monte Carlo settings
☒	☐	For hierarchical and complex designs, identification of the appropriate level for tests and full reporting of outcomes
☒	☐	Estimates of effect sizes (e.g. Cohen's *d*, Pearson's *r*), indicating how they were calculated

Our web collection on statistics for biologists contains articles on many of the points above.

Software and code

Policy information about availability of computer code

Data collection	Metabolite data was collected by an Orbitrap Exploris 120 LC–MS (Thermo Scientific, Pittsburgh, PA, USA) with Xcalibur v4.2.47 software. Gene expression data was collected by an iQ5 Real-Time PCR System (Bio-Rad, USA) with iQ5 Optical System v2.1 software.
Data analysis	Hidden Markov Model (HMM) for BAHD-ATs (PF02458) was used to identify the BAHD genes through the hmmsearch program against A. belladonna transcriptomes from the Medicinal Plant Genomics Resource (http://mpgr.uga.edu/) in HMMER 3.3.2. Batch CD-Search (https://www.ncbi.nlm.nih.gov/Structure/bwrpsb/bwrpsb.cgi) was used to further confirm that the candidate genes belonged to the BAHD-AT family. TBtools-II v2.042 was used to construct a the heatmap of the association analysis of the metabolites and BAHD gene family expression patterns was used to construct a heatmap of the relative expression level. Amino acid sequence alignment was performed using the E-INS-I method in MAFFT v7.475. The phylogenetic tree was constructed using IQ-TREE v2.1.2 with the maximum likelihood model (LG+F+G4). The tree was rooted using the algal enzyme clade (clade 0) and classified according to previous method. In addition, the tree was annotated and visualized with iTOL v6. GraphPad Prism 9.5.0 was use to draw Bar plots and box plots. GraphPad Prism 9.5.0 was used to fit Michaelis-Menten equation and caculate enzyme kinetic constant. All statistical analyses were conducted in GraphPad Prism 9.5.0. AlphaFold2 v2.3.0 was used for building the protein model of TS and predicting the substrate pocket. The ligands (3β-tropanol, acetyl-CoA, tigloyl-CoA and benzoyl-CoA) were downloaded from the PubChem database (https://pubchem.ncbi.nlm.nih.gov/) and docked into the cofactor-binding site of TS using AutoDock Tools v1.5.6. The interactions between the substrates and TS were analysed using PLIP v2.3.0. PyMOL 2.1 (http://www.pymol.org) was used to view the molecular interactions and process the image.

For manuscripts utilizing custom algorithms or software that are central to the research but not yet described in published literature, software must be made available to editors and reviewers. We strongly encourage code deposition in a community repository (e.g. GitHub). See the Nature Portfolio guidelines for submitting code & software for further information.

Data

Policy information about availability of data

All manuscripts must include a data availability statement. This statement should provide the following information, where applicable:

- Accession codes, unique identifiers, or web links for publicly available datasets
- A description of any restrictions on data availability
- For clinical datasets or third party data, please ensure that the statement adheres to our policy

The data used for transcriptome analysis comes from Medical Plant Genomics Resources (http://mpgr.uga.edu/). The sequences (46 BAHD acyltransferases of A. belladonna and 196 functionally identified BAHD acyltransferases) used to construct the phylogenetic tree are listed in Supplementary Data S1. Phylogenetic analysis of BAHD acyltransferases is shown in Supplementary Data S2. The 1H-NMR, 13 C-NMR, and 2D-NMR data of the chemicals synthesized in this study are listed in Supplementary Data S3. The coding sequence of TS has been uploaded to NCBI, and its accession number is OP677554. Source data are provided with this paper.

Research involving human participants, their data, or biological material

Policy information about studies with human participants or human data. See also policy information about sex, gender (identity/presentation), and sexual orientation and race, ethnicity and racism.

Reporting on sex and gender	N/A
Reporting on race, ethnicity, or other socially relevant groupings	N/A
Population characteristics	N/A
Recruitment	N/A
Ethics oversight	N/A

Note that full information on the approval of the study protocol must also be provided in the manuscript.

Field-specific reporting

Please select the one below that is the best fit for your research. If you are not sure, read the appropriate sections before making your selection.

☒ Life sciences ☐ Behavioural & social sciences ☐ Ecological, evolutionary & environmental sciences

For a reference copy of the document with all sections, see nature.com/documents/nr-reporting-summary-flat.pdf

Life sciences study design

All studies must disclose on these points even when the disclosure is negative.

Sample size	No sample size calculation was performed. All in vivo or in vitro experimental sample sizes are greater than or equal to 3 to meet the requirement of the two-sided independent sample t-test.
Data exclusions	No data were excluded from the analyses.
Replication	Three independent plants were used in the tissue profile analysis of 3β-tigloyloxytropane and the relative expression analysis of TS.<br>Fifteen independent plants each from the control group and VIGS-TS group were used in the VIGS assays.<br>In overexpression experiments, eight independently transformed root culture lines transformed with pBI121 was used as control (CK); OE denotes all independently transformed root culture lines overexpressing TS (three biological replicates for each line), including OE-1, OE-2, OE-3, and OE-4.<br>All tobacco transformation and microscopic analysis were independently conducted three times with different plant in this study.<br>Western blot analysis were independently conducted two times with different plant.<br>Recombinant protein obtained from three independent transformants of TS and each mutant for activity test.<br>In synthesizing of 3β-tigloyloxytropane in tobacco, leaves from three independent plants of each line was used for metabolite analysis.<br>In synthesizing of 3β-tigloyloxytropane in E. coli, three independent transformants of each line were used for analysis.
Randomization	Four lines with significantly increased expression of target genes (OE-1, OE-2, OE-3, and OE-4), independently transformed by the corresponding engineered vectors, were randomly selected for metabolite analysis.
Blinding	This is a study on plants, therefore the question is not of relevance.

Reporting for specific materials, systems and methods

We require information from authors about some types of materials, experimental systems and methods used in many studies. Here, indicate whether each material, system or method listed is relevant to your study. If you are not sure if a list item applies to your research, read the appropriate section before selecting a response.

Materials & experimental systems

n/a	Involved in the study
☐	☒ Antibodies
☒	☐ Eukaryotic cell lines
☒	☐ Palaeontology and archaeology
☒	☐ Animals and other organisms
☒	☐ Clinical data
☒	☐ Dual use research of concern
☐	☒ Plants

Methods

n/a	Involved in the study
☒	☐ ChIP-seq
☒	☐ Flow cytometry
☒	☐ MRI-based neuroimaging

Antibodies

Antibodies used

VDAC1 antibody (Orizymes, Cat#PAB220312); Goat Anti-Rabbit IgG H&L HRP (Biosharp, Cat#BL003A)

Validation

VDAC1 antibody (Orizymes, Cat#PAB220312)
http://www.phytoantibodies.com/a/ninanjie/qita/20220513/259.html
Host Organism: rabbit
Clonality: polyclonal
Dilution: 1:2000

Goat Anti-Rabbit IgG H&L HRP (Biosharp, Cat#BL003A)
http://www.biosharp.cn/index/product/details/language/cn/product_id/90.html
Host Organism: Goat
Clonality: polyclonal
Dilution: 1:5000

Dual use research of concern

Policy information about dual use research of concern

Hazards

Could the accidental, deliberate or reckless misuse of agents or technologies generated in the work, or the application of information presented in the manuscript, pose a threat to:

No	Yes
☒	☐ Public health
☒	☐ National security
☒	☐ Crops and/or livestock
☒	☐ Ecosystems
☒	☐ Any other significant area

Experiments of concern

Does the work involve any of these experiments of concern:

No	Yes	
☒	☐	Demonstrate how to render a vaccine ineffective
☒	☐	Confer resistance to therapeutically useful antibiotics or antiviral agents
☒	☐	Enhance the virulence of a pathogen or render a nonpathogen virulent
☒	☐	Increase transmissibility of a pathogen
☒	☐	Alter the host range of a pathogen
☒	☐	Enable evasion of diagnostic/detection modalities
☒	☐	Enable the weaponization of a biological agent or toxin
☒	☐	Any other potentially harmful combination of experiments and agents

Plants

Seed stocks	The seeds of Atropa belladonna and Nicotiana benthamiana used in this study were preserved in the Zhihua Liao Laboratory of Southwest University
Novel plant genotypes	There is no novel plant genotypes used in this study.
Authentication	The species authentication of Atropa belladonna and Nicotiana benthamiana was carried out by Hongping Deng, a plant taxonomist from Southwest University.
